# ParTIpy: a scalable framework for archetypal analysis and Pareto task inference

**DOI:** 10.1038/s44320-026-00209-6

**Published:** 2026-06-25

**Authors:** Philipp Sven Lars Schäfer, Leoni Zimmermann, Paul L Burmedi, Avia Walfisch, Noa Goldenberg, Shira Yonassi, Einat Shaer Tamar, Miri Adler, Jovan Tanevski, Ricardo O Ramirez Flores, Julio Saez-Rodriguez

**Affiliations:** 1 Institute for Computational Biomedicine, Heidelberg University and Heidelberg University Hospital, Heidelberg, Germany; 2 Department of Genetics, Silberman Institute of Life Sciences; and Department of Immunology and Cancer Research, Faculty of Medicine, The Hebrew University of Jerusalem, Jerusalem, Israel; 3 Department of Biological Chemistry, Silberman Institute of Life Sciences, The Hebrew University of Jerusalem, Jerusalem, Israel; 4 Center for Computational Medicine, The Hebrew University of Jerusalem, Jerusalem, Israel; 5 European Molecular Biology Laboratory, European Bioinformatics Institute (EMBL-EBI), Hinxton, Cambridgeshire UK

**Keywords:** Computational Biology

## Abstract

Trade-offs between different tasks are pervasive across scales in biological systems. For example, cells cannot perform all possible functions simultaneously; instead they allocate limited resources to specialize in subsets of tasks by activating specific gene expression programs. Pareto Task Inference (ParTI) is a framework for analyzing biological trade-offs grounded in multi-objective optimality. However, existing software for ParTI neither scales to large datasets nor integrates well with standard data analysis workflows. To address this gap, we developed ParTIpy (https://pypi.org/project/partipy), an open-source Python package that leverages optimization and coreset methods to scale archetypal analysis, the core algorithm underlying ParTI, to millions of cells. By providing tools to characterize archetypes and comprehensive documentation (https://partipy.readthedocs.io), ParTIpy integrates seamlessly into existing analysis workflows, especially for single-cell data. We demonstrate how ParTIpy can be used to study intra-cell-type gene expression variability through the lens of task allocation, offering a principled alternative to methods that impose discrete cell state classifications on inherently continuous variation.

## Synopsis

ParTIpy is a scalable, open-source Python framework for archetypal analysis that enables Pareto Task Inference at million-cell scale and provides an accessible, interoperable workflow for modeling intra-cell-type gene-expression variability as continuous task allocation rather than discrete cell states.

- ParTIpy is a highly scalable open-source Python framework for archetypal analysis, enabling Pareto Task Inference at million-cell scale with integrated model selection and stability diagnostics.
- Comprehensive documentation, tutorials, and scverse interoperability make Pareto Task Inference accessible to the broader single-cell community and beyond.
- Integrated tooling supports archetype characterization via enrichment analysis, ligand–receptor crosstalk inference, and projection of archetypal programs onto external reference datasets (e.g., spatial data).
- Establishes a workflow for cross-condition analysis of continuous shifts in cellular task allocation without imposing discrete cell-state boundaries.

## Abstract (web summary)

ParTIpy is a scalable, open-source Python framework for archetypal analysis that enables Pareto Task Inference at million-cell scale and provides an accessible, interoperable workflow for modeling intra-cell-type gene-expression variability as continuous task allocation rather than discrete cell states.

## Graphical abstract

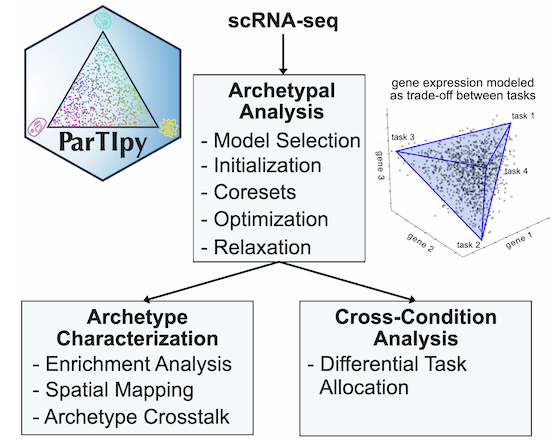

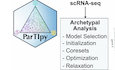

## Introduction

Trade-offs between different functions or tasks are pervasive across scales in biological systems. At the morphological level, the beaks of Darwin’s finches are a classical example: Their beaks cannot be optimal for both cracking seeds and picking insects, leading to specialization or compromise across species (Alon, 2020; Shoval et al, 2012). At the cellular scale, individual cells cannot perform all functions simultaneously; instead they allocate limited resources to specialize in subsets of tasks (Hart et al, 2015; Korem et al, 2015; Adler et al, 2019, 2023; Crowley et al, 2026). Pareto task inference (ParTI) is a framework for analyzing trade-offs grounded in the theory of multi-objective optimality (Shoval et al, 2012; Hart et al, 2015). It assumes that evolution drives phenotypes toward Pareto optimality, where improvement in one task necessarily worsens performance in another. Under the assumption of Pareto optimality, phenotypes (e.g., organisms, proteins, or cells), described by traits with trade-offs (e.g., beaks of finches, kinetic properties, or gene expression), lie within a polytope that has as many vertices as tasks being optimized (Shoval et al, 2012). In simple terms, a polytope is a shape with corners, straight edges, and flat faces, like a triangle or tetrahedron, but generalized to any number of dimensions. Leveraging this geometric structure, ParTI infers the underlying tasks by fitting a polytope to phenotypic measurements, where each vertex, referred to as an archetype, represents a phenotype optimized for a specific task. For example, in the context of single-cell transcriptomics, ParTI fits a polytope enclosing cells in gene expression space, with vertices (archetypes) representing cells optimized for distinct tasks. The gene expression profile of each archetype can then be analyzed to infer the specific tasks. The polytopes can be efficiently inferred using archetypal analysis, a dimensionality reduction technique that identifies extreme points of a dataset, corresponding to the archetypes, and models the dataset as nonnegative linear combinations of these archetypes (Cutler and Breiman, 1994; Mørup and Hansen, 2012; Alcacer et al, 2025).

Numerous studies have demonstrated the utility of archetypal analysis for modeling biological systems, particularly at the single-cell level (Thøgersen et al, 2013; Korem et al, 2015; Adler et al, 2019, 2023; Miyara et al, 2025; Mohammadi et al, 2018; Groves et al, 2022; Cook and Wrana, 2022; Hausser and Alon, 2020; Crowley et al, 2026), revealing trade-offs in cellular systems such as hepatocytes (Adler et al, 2019), fibroblasts (Adler et al, 2023), and cancer cells (Hausser and Alon, 2020). In addition, archetypal analysis has been applied to spatial transcriptomics and proteomics data, where instead of focusing on individual cells, the objective is to identify groups of spatially co-localized cells that cooperate to perform specific tasks, thus revealing a division of labor at the niche level (El Marrahi et al, 2023; Túrós et al, 2024; Kleshchevnikov et al, 2022; He et al, 2024).

To enable application of ParTI in biology, the ParTI MATLAB package (Hart et al, 2015) was developed, combining algorithms for polytope inference with tools for archetype characterization into a principled workflow for Pareto task inference. However, the software does not scale to large datasets, in particular those generated with high-throughput single-cell technologies. Moreover, it requires a commercial license (MATLAB), hindering broader adoption.

To address these limitations, we developed ParTIpy (Pareto Task Inference in Python), an open-source Python package that combines algorithmic innovations in initialization and optimization with the use of coresets (Mørup and Hansen, 2012; Bauckhage et al, 2015; Mair and Brefeld, 2019; Mair and Sjölund, 2024) to scale archetypal analysis to large-scale datasets. Although ParTIpy is broadly applicable, we focus here on its integration with single-cell analysis workflows in the scverse (Virshup et al, 2023). In particular, we show how ParTIpy can be used to study intra-cell-type variability through the lens of optimal resource allocation and division of labor, offering a principled alternative to approaches that impose discrete cell state classifications onto inherently continuous variation.

## Results

**Figure 1:**
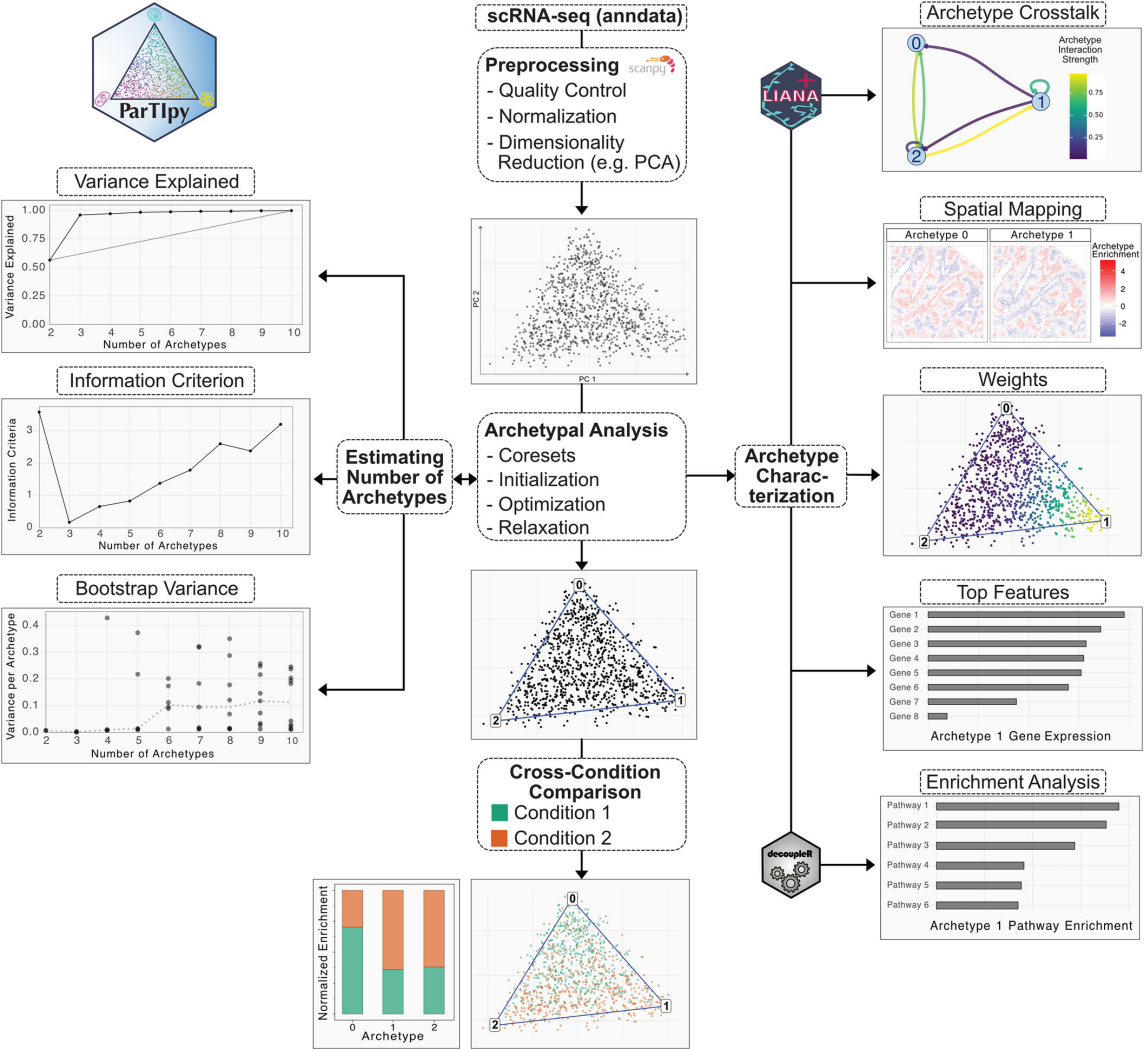
Overview of the ParTIpy framework. ParTIpy applies archetypal analysis to preprocessed single-cell data to represent gene expression as combinations of a few extreme transcriptional states, or archetypes. Each cell’s position relative to these archetypes reflects its bias toward one or more tasks. The workflow supports selection of the number of archetypes using variance explained, information criterion, and bootstrap stability, and provides functions for archetype characterization and cross-condition comparison. Archetype characterization comprises analyses that relate archetypes to biological features and contexts, including feature and pathway identification, spatial mapping, and crosstalk analysis.

ParTIpy provides a modular framework for applying archetypal analysis to multivariate data, with various options for initialization and optimization, and support for relaxing model constraints. It supports the use of coresets to scale analysis to large datasets and includes metrics to help determine the optimal number of archetypes. To support single-cell transcriptomic applications, ParTIpy integrates downstream functionalities for interpreting archetypes, including functional enrichment, cross-condition comparisons, spatial mapping, and inference of archetypal crosstalk networks via ligand–receptor co-expression analysis (Fig. 1).

### Archetypal analysis

Archetypal analysis finds K extreme points within the data distribution (convex hull of the data) called archetypes, such that all other data points can be described as mixtures (convex combinations) of these archetypes (Cutler an Breiman, 1994; Mørup and Hansen, 2012; Alcacer et al, 2025). Formally, the archetypes $Z\in {R}^{K\times D}$ are defined as convex combinations of the data points, $Z={BX}$ where $X\in {R}^{N\times D}$ is the data matrix with N cells, D features and $B\in {R}^{K\times N}$ is a nonnegative weight matrix where each row sums to one (row stochastic). This constraint ensures that the archetypes lie within the convex hull of the dataset *X*, forming *K* vertices of a polytope that approximates the data distribution. In the context of ParTI, each vertex corresponds to an inferred archetype that represents a distinct task a cell may be specialized in, reflecting an underlying division of labor. Each data point is then reconstructed as a convex combination of the archetypes, $X\approx {A\; Z}$ where $A{\in R}^{N\times K}$ is also row-stochastic. Putting everything together, archetypal analysis seeks a matrix *B* that defines *K* archetypes as extreme points within the data distribution, and a matrix *A* that reconstructs each data point as a convex combination of these *K* archetypes (see Eq. 1).

1 $$\begin{array}{c}\begin{array}{cc}\mathop{\min }\nolimits_{\begin{array}{c}A\in {{\mathbb{R}}}^{N\times K}\\ B\in {{\mathbb{R}}}^{K\times N}\end{array}}{\left|\left|{{X}-A \overbrace{{BX}}^{Z}}\right|\right|}_{F}^{2} & {\rm{subject}}\,{\rm{to}}\end{array} \\ \begin{array}{cc}A\ge 0, & A{1}_{K}={1}_{N}\\ B\ge 0, & B{1}_{N}={1}_{K}\end{array}\hfill\end{array}$$

However, when only a few cells have been sampled near the boundaries of the true phenotypic space, the convex hull of the observed data may underestimate the true range of variation. Such limited sampling near the boundaries can arise, for example, when the sample size is small or when the population is dominated by generalist cells rather than cells specialized in tasks. In these cases, relaxing the convexity constraint can help recover the true archetypes. This is achieved by introducing a relaxation parameter *δ* (see Eq. 2) (Mørup and Hansen, 2012), where larger values of *δ* permit archetypes to lie farther from the data cloud (see Appendix Fig. S1).

2 $$\begin{array}{c}\mathop{\min }\nolimits_{\begin{array}{c}A\in {{\mathbb{R}}}^{N\times K}\\ B\in {{\mathbb{R}}}^{K\times N}\\ \alpha \in {{\mathbb{R}}}^{K}\end{array}}\begin{array}{cc}{\left|\left|{{X}-A \overbrace{{\rm{diag}}(\alpha ){BX}}^{Z}}\right|\right|}_{F}^{2} & {\rm{subject}}\,{\rm{to}}\end{array}\\ \begin{array}{cc}A\ge 0, & A{1}_{K}={1}_{N}\end{array}\\ \begin{array}{c}\begin{array}{cc}B\ge 0, & B{1}_{N}={1}_{K}\quad\quad\quad\quad\end{array}\\ ({1}_{K}-\delta {1}_{K})\le \alpha \le \left({1}_{K}+\delta {1}_{K}\right)\end{array}\hfill\end{array}$$

**Figure 2:**
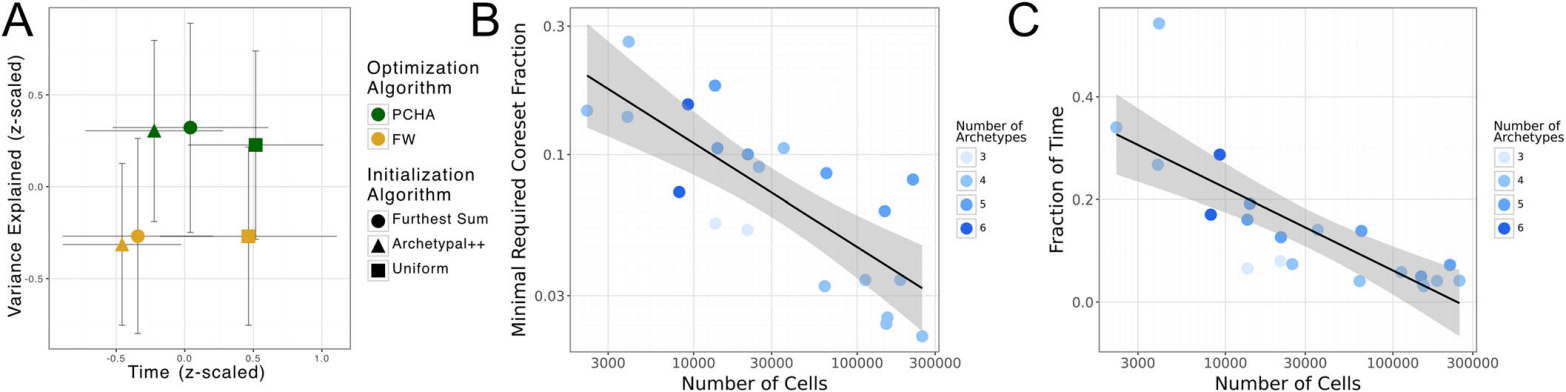
Benchmarking optimization parameters and coreset settings. (**A**) Summary of benchmark results for initialization and optimization algorithms across all datasets and cell types shown in Table EV1. Each data point is based on *N* = 21 runs; error bars denote mean ± standard deviation. (**B**) Minimal coreset size required to capture at least 95% of the variance explained by the full-data model. (**C**) Fraction of run time for optimization with the minimal required coreset size compared to the full-data model. In both panels, the solid line shows a linear regression fit based on *N* = 21 data points, and the shaded band indicates the pointwise 95% confidence interval of the fitted mean. Source data are available online for this figure.

The non-convexity of the objectives in Eqs. 1 and 2 gives rise to challenging optimization landscapes, where solution quality is highly sensitive to initialization. To address this, a range of initialization and optimization strategies have been developed to improve convergence and reconstruction accuracy (Alcacer et al, 2025). We have implemented and benchmarked three different initialization algorithms and two different optimization algorithms (see Appendix Supplementary Methods Sections 2.3 and 2.4). Using 21 single-cell transcriptome datasets, each corresponding to a single cell type, from three independent studies (Table EV1), we tested which combination of initialization and optimization algorithm offers the best trade-off between reconstruction accuracy and runtime (see Appendix Supplementary Methods Section 5.1). We found that using the principal convex hull analysis (PCHA) optimization algorithm (Mørup and Hansen, 2012) and the Archetypal++ (AA++) initialization (Mair and Sjölund, 2024) yields the best results (Fig. 2A; Appendix Fig. S2). However, if runtime is a limiting factor, the more efficient Frank-Wolfe (FW) algorithm (Bauckhage et al, 2015) provides a practical alternative.

To further improve the scalability of ParTIpy, we implemented an algorithm for constructing coresets specifically designed for archetypal analysis (see Appendix Supplementary Methods Section 2.5) (Mair and Brefeld, 2019). A coreset is a weighted subset of the data such that optimizing the objective on the coreset yields results that are close to those obtained using the full dataset. This requires incorporating a diagonal weight matrix *W* into the objective function, where $\widetilde{X}$ denotes the subsampled data matrix representing the coreset (Eq. 3).

3 $$\begin{array}{c}\mathop{\min }\limits_{\begin{array}{c}A\in {{\mathbb{R}}}^{N\times K}\\ B\in {{\mathbb{R}}}^{K\times N}\\ \alpha \in {{\mathbb{R}}}^{K}\end{array}}\begin{array}{cc}{\left|\left|{{W}\widetilde{X}-{WA} \overbrace{{\rm{diag}}(\alpha )B\widetilde{X}}^{Z}}\right|\right|}_{F}^{2} & {\rm{subject}}\,{\rm{to}}\end{array}\\ \begin{array}{cc}A\ge 0, & A{1}_{K}={1}_{N}\end{array}\quad\quad\quad\quad\quad\quad\quad\quad\quad\quad\quad\quad\\ \begin{array}{c}\begin{array}{cc}B\ge 0, & B{1}_{N}={1}_{K}\quad\quad\quad\quad\end{array}\\ ({1}_{K}-\delta {1}_{K})\le \alpha \le \left({1}_{K}+\delta {1}_{K}\right)\end{array}\hfill\end{array}$$

To support this formulation, we modified the optimization algorithms accordingly (see Appendix Supplementary Methods Section 2.5). Using the same datasets employed for benchmarking initialization and optimization strategies (see Table EV1), we evaluated the minimum coreset size required to achieve comparable results to full-data optimization under realistic conditions, defined as the smallest coreset capturing at least 99% of the variance explained by the full-data model. As shown in Fig. 2B,C and Appendix Fig. S4, we found that the required coreset fraction decreases with increasing dataset size. For instance, when analyzing datasets with over 100,000 cells, using only 1–10% of the data was sufficient on average, resulting in approximately a fourfold reduction in runtime.

Furthermore, we verified that coreset-based archetypes captured the same biological signal as full-data optimization. Specifically, we aligned coreset and full-data archetypes using Hungarian matching on pairwise Euclidean distances between archetype locations, and then compared the resulting archetype-specific gene-expression profiles. Across coreset fractions of >1% of cells, these profiles remained highly concordant, with a median Pearson’s correlation above 0.9 (Appendix Fig. S5).

We additionally quantified absolute wall-clock runtime and peak memory usage across dataset sizes from 10,000 to 2,000,000 cells on three large single-cell datasets (Appendix Fig. S6; Table EV3). For one million cells, fitting six archetypes on a 10% coreset required 45–105 s (mean across datasets), and coreset optimization consistently reduced runtime across archetype numbers (Appendix Fig. S6A,B). Peak memory scales mainly with the in-memory AnnData object, whereas the archetypal-analysis optimization step itself operates on a low-dimensional PCA representation and therefore requires comparatively little memory (Appendix Fig. S6C,D).

To validate that ParTIpy produces results comparable to the original ParTI MATLAB package (Hart et al, 2015), we analyzed three distinct single-cell datasets (see Table EV2) and compared the resulting archetypes. As shown in Appendix Fig. S3, we observed a high degree of concordance between the two implementations.

Altogether, ParTIpy offers a flexible and scalable implementation of archetypal analysis that supports various initialization and optimization schemes, includes convexity relaxation, and introduces coreset-based acceleration, extending the original ParTI framework while preserving result concordance.

### Choosing the number of archetypes

To determine the appropriate number of archetypes, we implemented three complementary criteria, each evaluated over a range of candidate values (see Fig. 1, left panel and Methods Section 5.6).

First, we compute the variance explained. An elbow in this curve suggests that adding additional archetypes yields diminishing returns. Second, we use an information-theoretic criterion (Suleman, 2017) where lower values indicate a favorable trade-off between model complexity (i.e., the number of archetypes) and variance explained. Third, we assess archetype robustness via bootstrapping: we apply archetypal analysis to multiple bootstrap samples, align the resulting archetypes across replicates, and compute the positional variance of each archetype (Hart et al, 2015; Korem et al, 2015). A lower mean variance indicates greater robustness.

### Characterizing the archetypes

**Figure 3:**
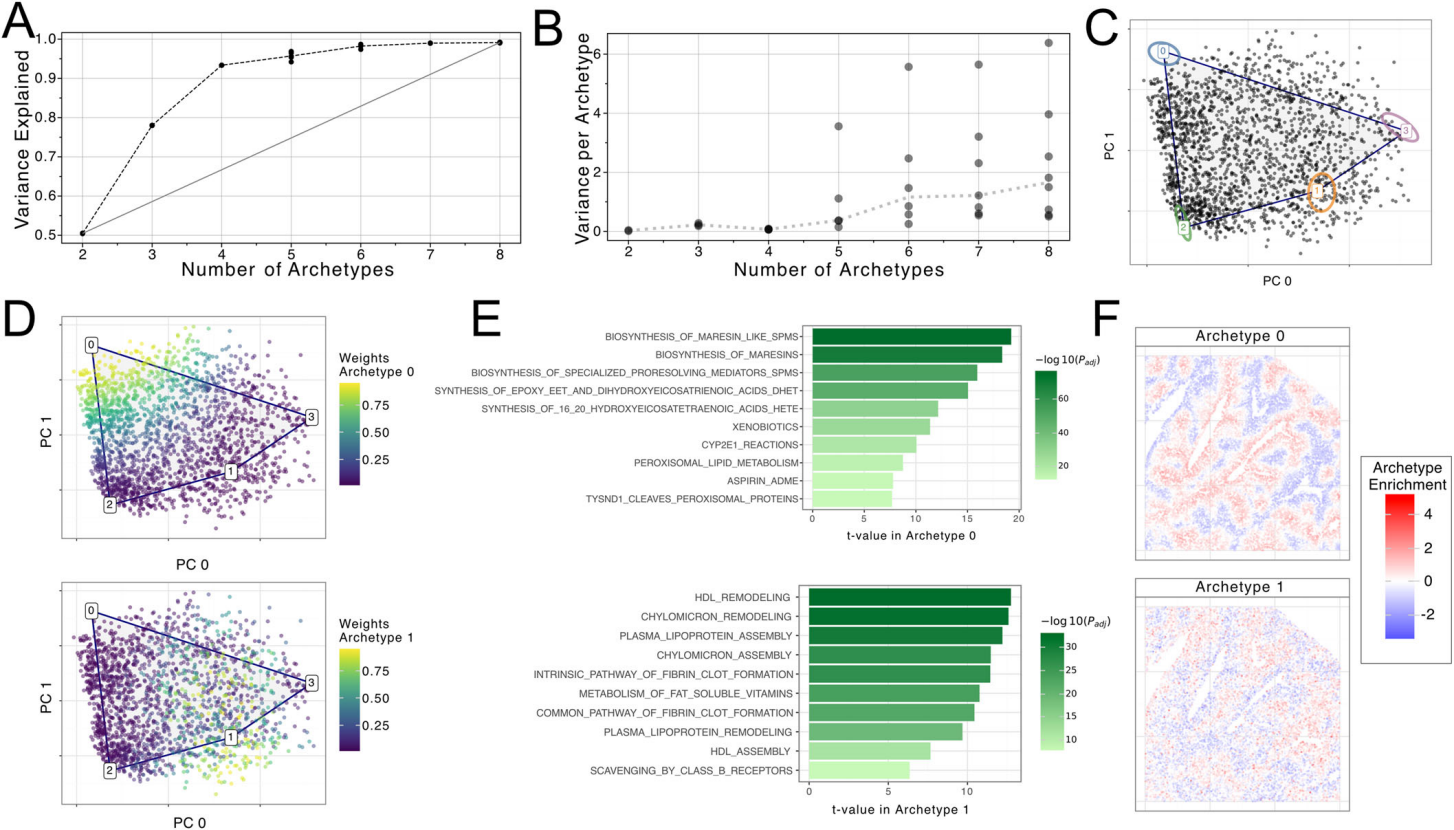
Example analysis on hepatocyte data from (Ben-Moshe et al, 2022). (**A**) Variance explained plotted for two to nine archetypes. (**B**) Bootstrap variance plotted for two to nine archetypes. (**C**) Two-dimensional PC plot showing single-cells in black, and the archetype in different colors; contours represent a 95% interval derived from the bootstrap samples. (**D**) Two-dimensional PC plot showing the weights used to characterize archetypes 0 and 1. (**E**) Barplots showing the top enriched Reactome pathways for archetypes 0 and 1. *t* values and *P* values are based on decoupler ULM run on archetype-aggregated (z-scored) expression profiles, reporting two-sided *p* values from the *t* statistic of the gene-set coefficient and BH-adjusted across Reactome pathways. (**F**) Spatial enrichment of archetypes 0 and 1. Source data are available online for this figure.

**Figure EV1:**
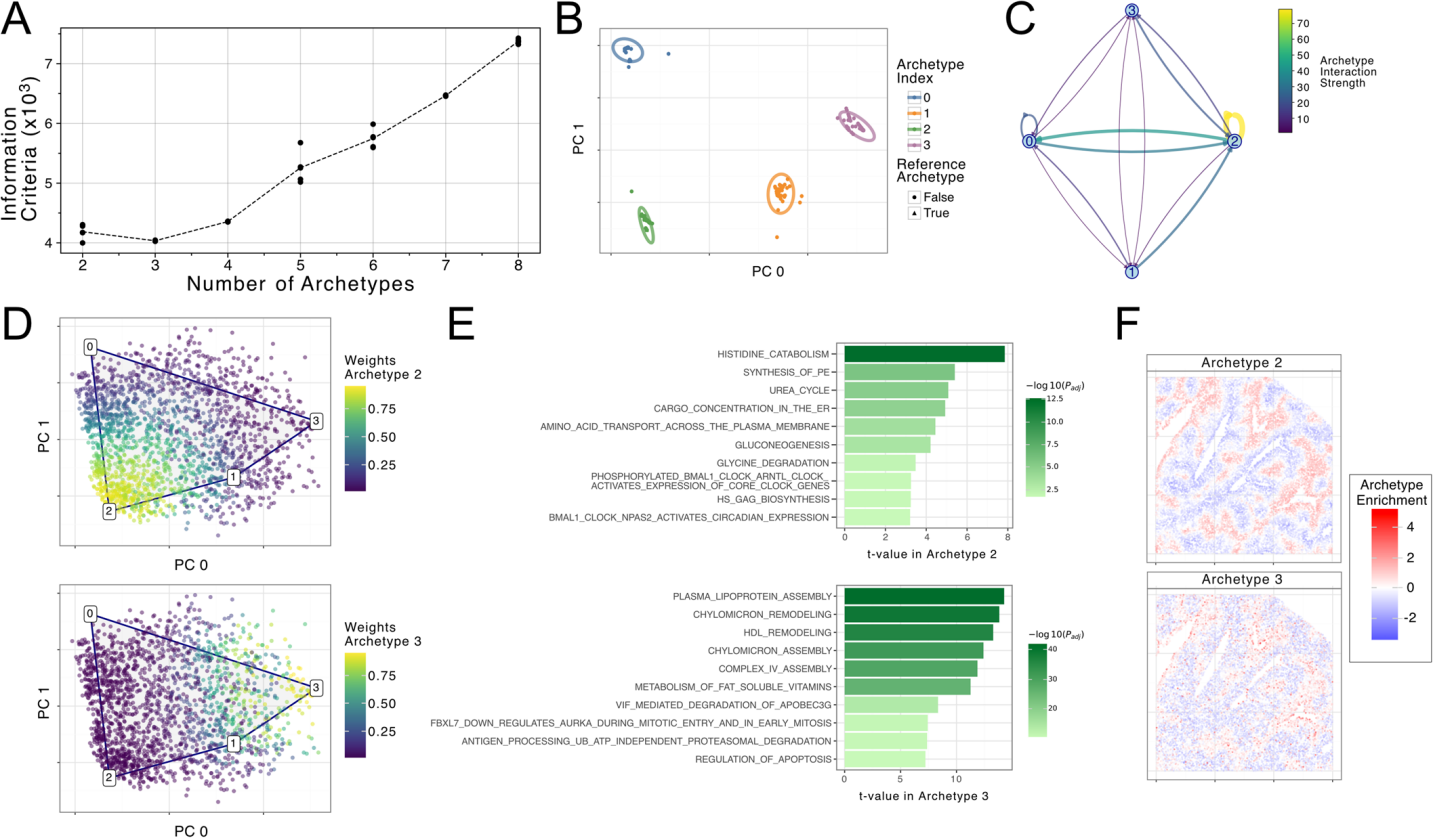
Example analysis on hepatocyte data from (Ben-Moshe et al, 2022). (**A**) Information criterion for archetypal analysis plotted for two to eight archetypes. (**B**) Two-dimensional PC plot showing the archetypal positions obtained from performing the optimization on 30 different bootstrap samples. (**C**) Crosstalk analysis shows strong self-interaction in archetype 2, and strong interaction between archetypes 0 and 2. (**D**) Two-dimensional PC plots showing the weights used to characterize archetypes 2 and 3. (**E**) Barplots showing the top enriched Reactome pathways for archetypes 2 and 3. *t* values and *P* values are based on decoupler ULM run on archetype-aggregated (z-scored) expression profiles, reporting two-sided p-values from the t-statistic of the gene-set coefficient and BH-adjusted across Reactome pathways. (**F**) Spatial mapping of archetypes 2 and 3. Source data are available online for this figure.

To characterize the archetypes, we first compute the distance of each cell to each archetype. We opted for distance-based characterization over direct use of the coefficients *A* or *B*. First, barycentric coordinates are not uniquely defined when the number of archetypes exceeds the embedding dimensionality plus one (e.g., four archetypes in a two-dimensional space), leading to non-identifiable cell–archetype coefficients. Second, direct characterization via the archetype-defining matrix *B* is impractical, as *B* is typically a sparse construction, which limits the stability and interpretability of aggregated feature profiles. These distances are then transformed into weights using a squared exponential kernel (see Figs. 3D and EV1D). By multiplying this weight matrix with the z-scored feature matrix, we obtain one aggregated feature profile per archetype (see Appendix Supplementary Methods Section 4). Alternatively, ParTIpy implements the quantile-based distance-ranking enrichment algorithm used in previous ParTI work (Korem et al, 2015; Adler et al, 2019; Hart et al, 2015) (see section “Quantile-based enrichment analysis”). This approach ranks cells by proximity to each archetype, partitions them into quantile bins, and tests whether gene expression is significantly elevated in the bin closest to the archetype relative to a defined background (by default, all other cells; using the Mann–Whitney *U*-test). While this procedure produces archetype-specific gene lists, it is computationally demanding because it requires repeated hypothesis testing for each gene–archetype pair. In practice, the kernel-based aggregation of z-scores described above yields highly similar gene rankings (see Appendix Fig. S7) while being substantially more time-efficient on large datasets.

For single-cell transcriptomic applications, archetypes can be characterized by directly inspecting the aggregated expression profiles or by performing pathway enrichment analysis. To facilitate the latter, ParTIpy seamlessly integrates with functions from decoupler-py (Badia-i-Mompel et al, 2022), enabling pathway enrichment analysis within the framework.

Having identified distinct archetypes and their associated biological functions, a natural next step is to ask what gives rise to this observed division of labor. One plausible explanation is the existence of spatial (chemical) gradients that render certain tasks more favorable in specific tissue regions (Adler et al, 2019). When spatial transcriptomic data from the same biological context are available, this hypothesis can be tested by mapping archetypes onto the spatial dataset, as described in Appendix Supplementary Methods Section 4.2. Another potential driver of functional specialization is cell–cell communication, such as lateral inhibition, where a cell performing a given task inhibits nearby cells from adopting the same task (Adler et al, 2023). To investigate whether such interactions underlie the observed division of labor, we infer potential signaling between archetypes by integrating their characteristic expression profiles with ligand–receptor databases (Dimitrov et al, 2024, 2022), following the workflow adapted from (Adler et al, 2023) (see Appendix Supplementary Methods Section 4.3).

### Using ParTIpy to characterize cellular task allocation

To illustrate the use of ParTIpy, we applied it to a single-cell RNA-seq dataset of hepatocytes sampled along the porto-central axis of the liver lobule (see Table EV2) (Ben-Moshe et al, 2022). Hepatocytes exhibit spatially constrained functional trade-offs, balancing distinct metabolic, biosynthetic, and detoxification processes (Halpern et al, 2017), and ParTI has previously been used to infer archetypes related to this division of labor (Adler et al, 2019). We first used the three-archetype selection criteria introduced above to determine the number of archetypes (Figs. 3A–C and EV1A). Based on these metrics, we selected four archetypes, as increasing beyond four yielded negligible gains in variance explained and led to reduced robustness. To characterize the resulting archetypes, we performed pathway enrichment analysis using decoupler-py (Badia-i-Mompel et al, 2022) and the Reactome database (Gillespie et al, 2022). Archetype 0, for example, was associated with drug and xenobiotic metabolism, while archetype 1 was enriched for lipid metabolism (Fig. 3E; Appendix Fig. S4J–M). To investigate potential mechanisms underlying this division of labor, we mapped the archetypes onto single-cell resolved spatial transcriptomics data (Vizgen MERFISH Mouse Liver Map January 2022). This analysis revealed that archetype 0 is localized near the central vein, whereas archetype 1 is enriched near the portal node (see Fig. 3F), consistent with previous hepatocyte archetype analysis findings (Adler et al, 2019) and established zonation patterns (Halpern et al, 2017). These results suggest that the observed functional specialization is driven by spatial gradients of oxygen and other metabolites along the porto-central axis.

### Using ParTIpy to quantify cross-condition shifts in cellular task allocation

Cross-condition analyses are a central component of single-cell transcriptomic studies and are commonly performed by discretizing cells into clusters followed by tests for condition-dependent changes in cluster abundance or gene expression. While effective in many settings, such approaches impose sharp boundaries on cell states that are often intrinsically continuous. ParTIpy enables an alternative formulation by modeling cells as continuous mixtures of archetypal programs (“tasks”), thereby enabling the quantification of condition-dependent transcriptional shifts within a shared task space.

**Figure 4:**
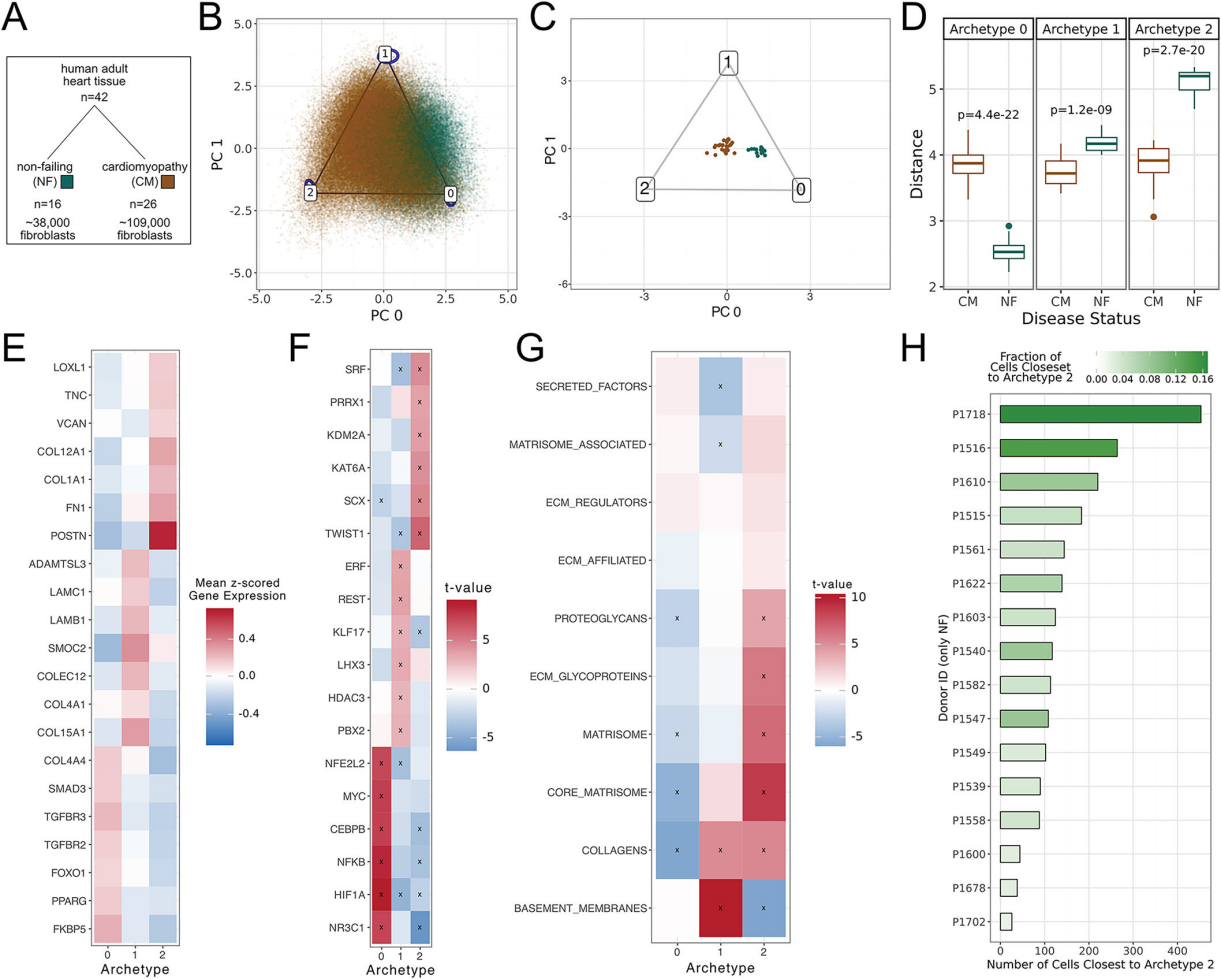
Cross-condition archetypal analysis of human cardiac fibroblasts in non-failing hearts and cardiomyopathy. (**A**) Study design and sample composition. (**B**) Archetypal analysis (AA) fit shown in the first two harmony-integrated principal components. Each point denotes a fibroblast nucleus, colored by disease status. The triangle indicates the inferred K = 3 archetypes, representing extreme transcriptional programs that span the observed continuous fibroblast state space. (**C**) Donor-level pseudobulk profiles in the same PC space and shown within the archetype convex hull, illustrating condition-dependent shifts at the patient level. (**D**) Donor-level distances to each archetype stratified by disease status. Boxplots summarize donor distributions; distances quantify proximity of each donor’s pseudobulk profile to each archetypal program (smaller distance indicates closer proximity). In each boxplot, the center line denotes the median, the box bounds denote the 25th and 75th percentiles, and the whiskers extend to the most extreme data points within 1.5 × the interquartile range (IQR) from the lower and upper quartiles, respectively; points beyond the whiskers are shown individually as outliers. Sample sizes: non-failing (NF), *n* = 16 donor hearts; cardiomyopathy (CM), *n* = 26 donor hearts. *P* values shown are from two-sided Welch’s *t*-tests comparing CM vs non-failing donor distances for each archetype. (**E**) Expression of curated fibroblast marker genes across archetypes, shown as mean z-scored expression in archetype-aggregated profiles, highlighting archetype-specific ECM remodeling and perivascular/basement-membrane programs. (**F**) Transcription factor (TF) activity differences across archetypes inferred using the univariate linear model (ULM) implemented in decoupler (Badia-i-Mompel et al, 2022) with the CollecTRI regulon resource (Müller-Dott et al, 2023). ULM estimates TF activity and derives two-sided *p*-values from a linear model relating archetype-aggregated expression to TF–target weights. *P*-values are adjusted for multiple testing using the Benjamini–Hochberg procedure; significance marks indicate *p*_adj ≤0.05. (**G**) Matrisome program activity across archetypes inferred using ULM with MSigDB NABA Matrisome gene sets (Naba et al, 2016). *P* values are derived using the ULM framework described in (**F**). (**H**) Number of cells per non-failing donor that are closest to archetype 2. Source data are available online for this figure.

To illustrate this workflow, we analyzed single-nucleus RNA-seq data from human adult heart tissue comprising 16 non-failing (NF) donor hearts and 26 cardiomyopathy (CM; pooled hypertrophic and dilated cardiomyopathy) hearts, focusing on approximately 147,000 fibroblasts (Fig. 4A) (Chaffin et al, 2022). Fibroblasts are particularly well suited for this analysis, as they exhibit pronounced disease-associated transcriptional remodeling and span a continuum from quiescent ECM maintenance to activated, fibrotic programs in cardiomyopathy (Patrick et al, 2024; Lanzer et al, 2025; Youness et al, 2025).

**Figure EV2:**
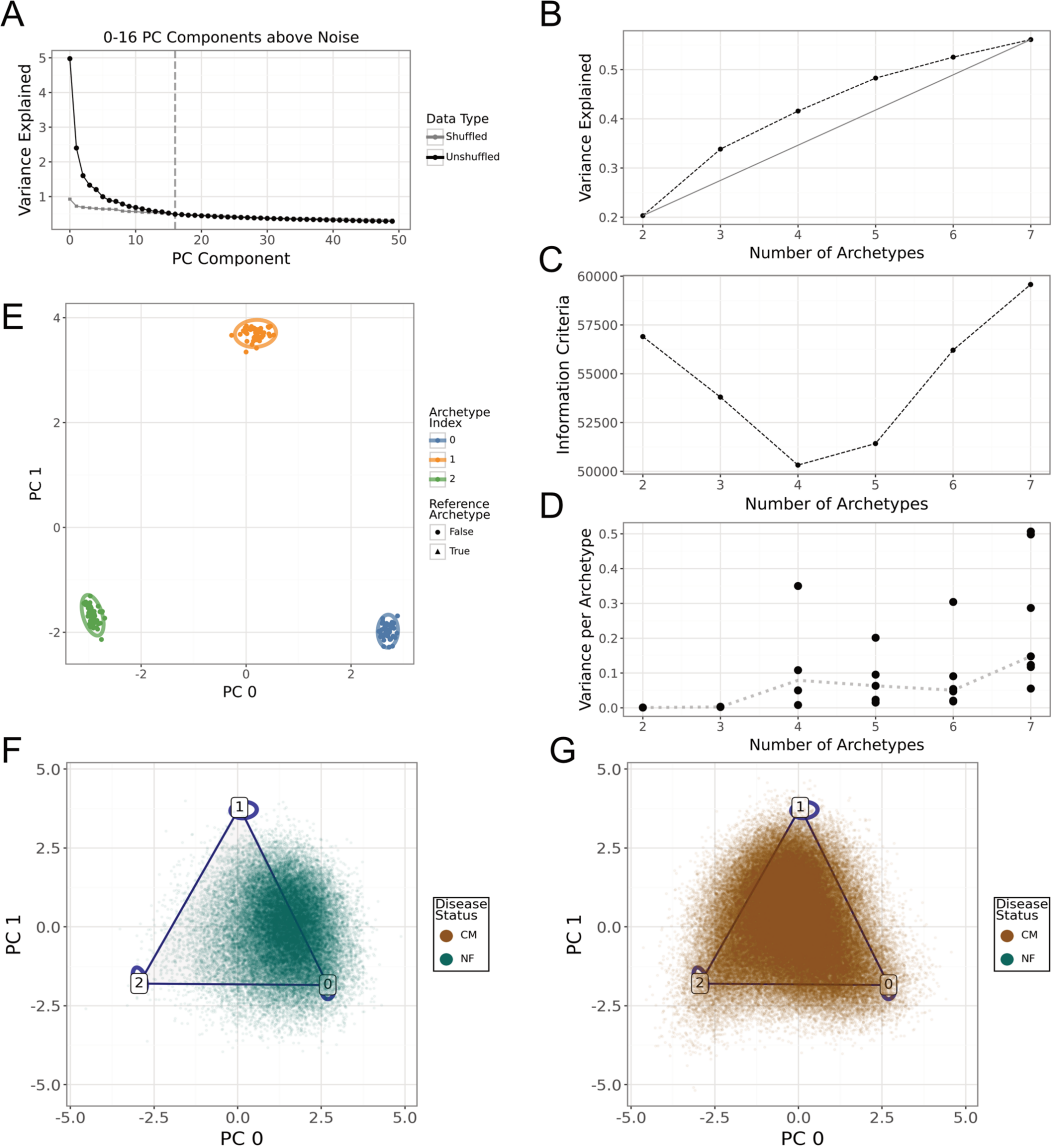
Model selection and robustness diagnostics for the fibroblast cross-condition analysis. (**A**) Shuffled principal component (PC) analysis used to determine the dimensionality of the input space for archetypal analysis. Variance explained by PCs from the original data (black) is compared to shuffled data (gray); PCs exceeding the shuffled baseline (PCs 0–16) were retained for downstream analysis. (**B**) Variance explained by archetypal analysis as a function of the number of archetypes (K = 2–7). (**C**) Information criterion for archetypal analysis evaluated across archetype numbers. (**D**) Bootstrap-based archetype stability analysis. Points show positional variance of individual archetypes across bootstrap replicates for each K; the dashed line indicates the mean variance across archetypes. Lower variance corresponds to more stable archetype solutions. (**E**) Two-dimensional PC projection showing the locations of archetypes inferred across bootstrap replicates for K = 3. Ellipses indicate the dispersion of bootstrap archetype positions around the reference archetypes, illustrating high positional stability for K = 3. (**F**, **G**) Two-dimensional PC projection of single-cell fibroblast profiles colored by disease status (NF and CM), with the inferred three-archetype simplex overlaid. Both conditions populate the same archetypal geometry, while disease-associated shifts are evident as changes in cell density toward specific archetypal programs. Source data are available online for this figure.

Following normalization, dimensionality reduction, and donor-level batch correction using Harmony (Korsunsky et al, 2019) (see Methods), we determined the dimensionality of the archetypal analysis input using a shuffled PCA baseline (see Methods) and retained the first 16 principal components (Fig. EV2A). We then evaluated the number of archetypes using the complementary criteria implemented in ParTIpy (Fig. EV2B–D). While both the variance explained and the information criterion favored a four-archetype solution, this configuration yielded one archetype with high positional variance across bootstrap replicates, indicating limited robustness. We therefore selected a three-archetype model, which showed reduced reconstruction but substantially improved bootstrap stability and reproducibility, enabling a more robust and interpretable biological characterization (Figs. 4B and EV2D,E).

To quantify the shift in task allocation during cardiomyopathy, we aggregated cells into donor-level pseudobulks by averaging the Harmony-corrected principal component coordinates of all fibroblasts within each donor (Fig. 4C). This avoids pseudo-replication and provides a concise summary of the mean fibroblast task allocation per patient. To formally assess these differences, we computed Euclidean distances from each donor-level pseudobulk to each archetype and compared distances between cardiomyopathy and non-failing donors using two-sided Welch’s t-tests. Distances to all three archetypes differed significantly between conditions (Fig. 4D). These differences were most pronounced for archetype 0, which was closer to non-failing donors, and archetype 2, which was closer to cardiomyopathy donors, whereas shifts along archetype 1 were comparatively weaker.

To interpret the biological meaning of the inferred archetypes, we characterized their transcriptional profiles using the aggregated z-scored gene expression together with downstream estimation of transcription factor activities and gene-set enrichment analyses. Archetype 2, which was preferentially enriched in cardiomyopathy donors, corresponded to an activated fibrotic program, marked by relatively higher expression of canonical fibrotic ECM genes including POSTN, FN1, COL1A1, VCAN, TNC, and LOXL1 (Fig. 4E) (Torimoto et al, 2024; Fu et al, 2018). Consistently, this archetype showed strong activation of mesenchymal and mechanotransduction-associated transcription factors such as TWIST1 (Wei et al, 2015), SCX (Bagchi et al, 2016), PRRX1 (Lee et al, 2022), and SRF (Olson and Nordheim, 2010), as inferred from CollecTRI (Müller-Dott et al, 2023) regulons using decoupler ULM (Badia-i-Mompel et al, 2022) (Fig. 4F). At the pathway level, archetype 2 was enriched for MSigDB NABA Matrisome gene sets (Naba et al, 2016) related to the core matrisome, collagens, ECM glycoproteins, and proteoglycans, reflecting broad extracellular matrix remodeling (Fig. 4G).

In contrast, archetype 1 was characterized by a perivascular and basement-membrane–associated program, with elevated expression of structural ECM components such as COL15A1, COL4A1, and laminins (including LAMB1 and LAMC1) (Gatseva et al, 2019), and comparatively limited activation of fibrotic genes (Fig. 4E). This archetype showed selective enrichment of NABA gene sets associated with basement membranes and ECM-affiliated proteins, consistent with a more structural and niche-supporting fibroblast role (Fig. 4G).

Archetype 0, which was preferentially enriched in non-failing donors, exhibited features of a quiescent, yet stress-responsive program (Chen et al, 2012). This archetype was characterized by genes involved in hypoxic, inflammatory, and oxidative stress responses and was associated with increased activity of stress-associated transcription factors, including NFKB, HIF1A, NR3C1, CEBPB, and NFE2L2 (Fig. 4F). Correspondingly, matrisome enrichment for archetype 0 was limited and depleted from core fibrotic components (Fig. 4G).

Although archetype 2 was preferentially enriched in cardiomyopathy, it was not specific to that condition. In every non-failing donor, a relatively small subset of fibroblasts was closer to archetype 2 than to any other archetype (median 115 cells per donor; Fig. 4H). Together, these results indicate that cardiomyopathy redistributes cardiac fibroblasts from stress-responsive toward activated fibrotic programs within a shared archetypal space, reflecting altered allocation among existing cellular programs rather than the emergence of disease-specific fibroblast states. More broadly, this example illustrates how ParTIpy enables a principled and interpretable framework for cross-condition analysis in single-cell data by linking the low-dimensional geometric structure of gene expression in single cells to biologically meaningful shifts in cellular task allocation.

## Discussion

Here we present ParTIpy, an open-source Python package that scales archetypal analysis by implementing state-of-the-art initialization and optimization techniques in combination with coresets, enabling the application of ParTI to large datasets, for example, the ones generated by modern single-cell transcriptomics technologies (Zhang et al, 2025). Applied to single-cell data, ParTIpy reveals how individual cells distribute across competing transcriptional programs, offering a complementary perspective to conventional clustering approaches. While we mainly presented examples on dissociated single-cell data, ParTIpy is broadly applicable to other biological contexts; for example, our online tutorials demonstrate its use with spatial transcriptomics.

ParTIpy implements two optimization algorithms for archetypal analysis (Alcacer et al, 2025), selected from the broader collection for their flexibility and efficiency, and our benchmarks and applications confirm that they provide the scalability required for modern single-cell datasets. Our gradient-based optimization algorithms readily accommodate modifications to the objective function, including the convexity relaxation that allows archetypes to extend beyond the data cloud. One notable extension of archetypal analysis is deep archetypal analysis (Milite et al, 2025; van Dijk et al, 2019; Wang and Zhao, 2022; Keller et al, 2019), which replaces the direct optimization of coefficient matrices with an encoder-decoder network. The encoder learns a posterior distribution for the latent representation of the data (*X*) and for the coefficient matrices *A,B*, which define the archetypes $\left(Z={BX}\right)$ and reconstruction $\left(\hat{X}={AZ}\right)$. This approach captures non-linear structures in the data and achieves scalability to large datasets through mini-batch optimization and amortized variational inference. While amortized inference provides a direct mapping from observations to archetypal coordinates, enabling fast inference once the model is trained, it can introduce an amortization gap, so that the predicted archetypal coordinates are suboptimal compared to those obtained by directly optimizing variational parameters (Margossian and Blei, 2024; Cremer et al, 2018). In contrast, ParTIpy achieves scalability through coreset-based optimization, enabling direct parameter inference even for large datasets. Furthermore, ParTIpy adopts linear dimensionality reduction, which ensures that the archetypes correspond to extremal points in the original trait space, consistent with the theoretical foundations of ParTI.

ParTIpy’s scalability, downstream utilities, and scverse interoperability, enable Pareto task inference in modern cross-condition single-cell studies, where differences between conditions can be interpreted as shifts in the usage of a shared set of archetypes (tasks). This application provides a continuous view of condition-associated task reallocation. When snapshot data capture ongoing cellular transitions (e.g., tissue remodeling), or when densely sampled time-course data were available, integrating estimated cell–cell transition probabilities (Weiler et al, 2024)—derived from RNA velocity, pseudotime algorithms, or optimal transport—could further strengthen interpretation by distinguishing stable task specializations from transient states and by quantifying trajectories within the archetypal simplex (Liu et al, 2025).

In conclusion, ParTIpy combines computational efficiency with biological interpretability in a versatile framework for exploring functional trade-offs in high-dimensional biological data. With dedicated tools for archetype characterization, seamless integration into Python-based workflows, and comprehensive documentation (https://partipy.readthedocs.io), it broadens access to ParTI for the research community.

## Methods

**Table:** Reagents and tools table

Reagent/resource	Reference or source	Identifier or catalog number
**Software**		
python v3.11	https://anaconda.org/conda-forge/python	conda-forge::python = =3.11
scanpy v1.11.1	https://anaconda.org/channels/conda-forge/packages/scanpy/overview	conda-forge::scanpy = =1.11.1
numpy v2.1.3	https://anaconda.org/conda-forge/numpy	conda-forge::numpy = =2.1.3
scipy v1.15.2	https://anaconda.org/conda-forge/scipy	conda-forge::scipy = =1.15.2
numba v0.61.0	https://anaconda.org/conda-forge/numba/	conda-forge::numba = =0.61.0
pandas v2.2.3	https://anaconda.org/conda-forge/pandas	conda-forge::pandas = =2.2.3
matplotlib v3.10.1	https://anaconda.org/conda-forge/matplotlib	conda-forge::matplotlib = =3.10.1
plotnine v0.14.5	https://anaconda.org/conda-forge/plotnine	conda-forge::plotnine = =0.14.5
plotly v6.1.1	https://anaconda.org/conda-forge/plotly/	conda-forge::plotly = =6.1.1
harmonypy v0.2.0	https://pypi.org/project/harmonypy/	pip: harmonpy = =0.2.0
decoupler v2.0.2	https://pypi.org/project/decoupler/	pip: decoupler = =2.0.2
liana v1.5.1	https://pypi.org/project/liana/	pip: liana = =1.5.1
scikit-misc v0.5.1	https://pypi.org/project/scikit-misc/	pip: scikit-misc = =0.5.1
ParTIpy	https://github.com/saezlab/ParTIpy	pip: partipy = =0.2.0
py_pcha	https://github.com/psl-schaefer/py_pcha	commit: cc819530defde37a5850e164a17ac9138b62ef21

### Archetypal analysis

Archetypal analysis (AA) is a matrix factorization framework that represents each observation as a convex combination of a small number of extreme points, termed archetypes, which themselves lie within (or near) the convex hull of the data (Cutler and Breiman, 1994; Mørup and Hansen, 2012; Alcacer et al, 2025). Given a data matrix $X\in {R}^{N\times D}$, archetypes $Z\in {R}^{K\times D}$ are defined via $Z={BX}$, where $B\in {R}^{K\times N}$ is nonnegative and row-stochastic. Each data point is then approximated as $X\approx {AZ}$, with $A\in {R}^{N\times K}$ also non-negative and row-stochastic. This geometric formulation yields a convex polytope spanned by the archetypes, providing an interpretable description of continuous variation in high-dimensional data. The full mathematical details are described in Section 2 of the Appendix Supplementary Methods.

In ParTIpy, archetypal analysis is applied to a low-dimensional representation of the data (typically PCA or an integrated PCA space after batch correction). Because the resulting optimization problem is non-convex (see Appendix Supplementary Methods 2.2), solution quality depends on both initialization and optimization strategy. ParTIpy implements multiple initialization schemes: Uniform Random, Furthest Sum (Mørup and Hansen, 2012), and archetypal analysis++ (AA++) (Mair and Sjölund, 2024); and multiple optimization algorithms: Principal convex hull analysis (PCHA) (Mørup and Hansen, 2012), a Frank-Wolfe (FW) variant (Bauckhage et al, 2015), and the original regularized nonnegative least squares (RNNLS) (Cutler and Breiman, 1994) (see Appendix Supplementary Methods Sections 2.3 and 2.4).

To enable scalable archetypal analysis on large datasets, ParTIpy supports coreset-based optimization, in which archetypal analysis is performed on a weighted subset of the data that approximates the full-data objective (see Appendix Supplementary Methods Section 2.5). Coreset weights are incorporated directly into the objective function as described in Eq. 3.

### Benchmarking optimization and initialization algorithms

Benchmarking was performed on 21 single-cell transcriptomic datasets, each representing a single annotated cell type, compiled from three independent studies spanning two diseases and two spatial/transcriptomic technologies: (i) snRNA-seq of post-mortem white-matter samples from chronic active and inactive multiple-sclerosis (MS) lesions and controls (52), comprising six major CNS cell classes (astrocytes, oligodendrocytes, oligodendrocyte progenitors, myeloid cells, endothelial cells, and neurons); (ii) 10x Xenium spatial transcriptomics of post-mortem spinal-cord multiple-sclerosis lesions and controls (51), covering seven cell types including immune, glial, and vascular populations; and (iii) scRNA-seq of peripheral blood mononuclear cells (PBMC) from systemic lupus erythematosus patients and healthy controls (53), encompassing eight immune cell types (Table EV1).

For all datasets, preprocessing of data was done with scanpy (Wolf et al, 2018). We applied total-count normalization (sc.pp.normalize_total), log1p transformation (sc.pp.log1p), selection of highly variable genes (sc.pp.highly_variable_genes), and principal component analysis computed on the selected HVGs (sc.pp.pca with mask_var = “highly_variable”). In contrast, the MS Xenium datasets were processed without HVG selection, as the number of measured genes was limited. Across all benchmarks, the first ten principal components were used as the archetypal analysis input. All Initialization algorithms were tested, but only PCHA and FW were tested as optimization algorithms. RNNLS was excluded from the benchmarked optimization set due to poor scalability. Benchmarks were iterated over multiple random seeds (MS snRNA-seq: 40 seeds; MS Xenium: 20 seeds; lupus PBMC: 20 seeds). For each init/optim/seed combination, archetypes were fit with n_restarts = 1, and runtime was measured using wall-clock time around the fitting call. For each run, the residual sum of squares (RSS) and variance explained were recorded and aggregated into z-scored summaries for cross-setting comparisons.

### Benchmarking coresets

Coreset benchmarking was performed on the same datasets used for the initialization and optimization benchmarks (see section “Benchmarking optimization of initialization algorithms”). We evaluated coreset fractions of 0.64, 0.32, 0.16, 0.08, 0.04, 0.02, 0.01, and 0.005.

For each dataset, reference archetypes were computed on the full data using five random restarts (n_restarts = 5) with a fixed random seed (42). Using these reference archetypes, we computed archetype weights and z-scaled archetype expression profiles. For each coreset fraction and random seed (MS snRNA‑seq: 20 seeds; MS Xenium: 20 seeds; lupus PBMC: 20 seeds), archetypal analysis was then performed using the default coreset algorithm (coreset_algorithm = “standard”).

Archetypes inferred from each coreset run were aligned to the corresponding reference archetypes using Hungarian matching based on pairwise Euclidean distances between archetype locations. To quantify agreement between coreset- and full-data solutions, we computed the Pearson correlation between archetype-specific characteristic expression profiles, as well as the variance explained by the archetypal reconstruction.

To estimate the minimal coreset fraction required to recover the full-data solution, we fitted a generalized additive model (GAM) with a B-spline basis to the relationship between variance explained and log10-transformed coreset fraction. We then selected the smallest coreset fraction whose predicted variance explained was within 1% of the variance explained by the full-data model.

Finally, runtime savings were quantified by fitting a second GAM to wall-clock runtime as a function of log10 coreset fraction and comparing the full-data runtime to the predicted runtime at the minimal coreset fraction.

### Benchmarking memory and time requirements

Memory and runtime benchmarks were performed on three large single-cell datasets (K562, HCT116, and HEK293T) (Appendix Fig. S6; Table EV3). To reduce variability due to parallelization, BLAS thread counts were fixed to one for all benchmark runs.

Peak memory usage was measured using resident set size (RSS), tracked via /proc/self/statm and ru_maxrss, with sampling every 50 ms. To assess scaling behavior, datasets were subsampled to predefined numbers of cells using a fixed random seed (42) to generate reproducible index subsets. Subsampling ranges were 1000 to 1,000,000 cells for K562 and 1000 to 2,000,000 cells for HCT116 and HEK293T. Each subsample was processed independently.

Preprocessing consisted of total-count normalization, log1p transformation, selection of highly variable genes (n_top_genes = 2000), and principal component analysis (PCA). The first 20 principal components were used as the input embedding for archetypal analysis.

Benchmarks were conducted across combinations of the number of archetypes $\left(K\in \mathrm{3,6,9}\right)$, coreset fractions (0.1,1.0), and five random seeds. Each benchmark run was executed in a separate process. For runs with coreset_fraction <1.0, the standard coreset algorithm was used.

To isolate the memory footprint of the archetypal analysis optimization step itself, we performed an additional PCA-only benchmark in which archetypal analysis was applied directly to precomputed PCA matrices rather than full AnnData objects. Specifically, we extracted the PCA embedding (pca_bench=adata.obsm[“X_pca”][:, :20]) and fit an archetypal analysis model directly on this matrix using pt.AA(…).fit(X=pca_bench), instead of invoking pt.compute_archetypes on an AnnData object. This benchmark excludes memory overhead associated with having the full single-cell AnnData object in memory, thereby attributing observed memory usage specifically to the optimization procedure operating on the low-dimensional embedding.

### Comparing ParTIpy and ParTI

To validate that ParTIpy produces results comparable to the original ParTI MATLAB package (Hart et al, 2015), we analyzed three distinct single-cell datasets (see Table EV2) and compared the resulting archetypes for *δ* = 0.0 and δ=0.5. For all datasets, preprocessing of data were done with scanpy (56). We applied total-count normalization (sc.pp.normalize_total), log1p transformation (sc.pp.log1p), selection of highly variable genes (sc.pp.highly_variable_genes), and principal component analysis computed on the selected HVGs (sc.pp.pca with mask_var = “highly_variable”). For both ParTIpy and ParTI, the default settings were used. Because archetype labels are not identifiable across runs, ParTI and ParTIpy archetypes were aligned in PCA space using Hungarian matching with pairwise Euclidean distances as the cost.

### Choosing the number of archetypes

There is no universally optimal method for selecting the number of archetypes *K* (Alcacer et al, 2025). In practice, *K* is chosen using a combination of complementary heuristics that balance reconstruction accuracy, model complexity, and robustness. Below, we describe the criteria implemented in ParTIpy.

For a fixed number of archetypes *K*, reconstruction quality is quantified by the fraction of variance explained, defined as

$${R}^{2}=\frac{{\rm{TSS}}-{\rm{RSS}}}{{\rm{TSS}}}=1-\frac{{{||X}-{ABX||}}_{F}^{2}}{{{||X||}}_{F}^{2}},$$

where *X* denotes the input data matrix and *A* and *B* are the optimized archetypal coefficient matrices. As *K* increases, *R*^2^ typically exhibits diminishing returns; an elbow in the *R*^2^-vs-*K* curve indicates that additional archetypes provide limited improvement in reconstruction accuracy.

To explicitly penalize model complexity, ParTIpy additionally reports an information-theoretic criterion adapted for archetypal analysis (Suleman, 2017), defined as

$${{\rm{v}}}_{{\rm{AA}}}=\log \left(\frac{1}{{\rm{ND}}}{{||}{\rm{X}}-\hat{{\rm{X}}}{||}}_{{\rm{F}}}^{2}\right)+2\frac{{{\rm{K}}}_{{\rm{a}}}+{{\rm{K}}}_{{\rm{b}}}+1}{{\rm{N}}{\rm{tr}}\left({\Sigma }_{\hat{{\rm{X}}}}{\Sigma }_{{\rm{X}}}^{-1}\right)},$$

where $\hat{X}={ABX}$ is the reconstructed data, *N* is the number of observations, *D* the dimensionality, *K*_*a*_ = *N* (*K*-1) and ${K}_{b}=K\left(N-1\right)$ denote the effective numbers of free parameters in *A* and *B*, respectively, and ${\varSigma }_{\hat{X}}$ and ${\varSigma }_{X}$ are the empirical covariance matrices of the reconstructed and original data. Smaller values of ${v}_{{AA}}$ indicate a more favorable trade-off between goodness of fit and model complexity.

Finally, the robustness of inferred archetypes is assessed using bootstrap-based stability analysis. For a fixed *K*, reference archetypes ${z}_{1},\ldots ,{z}_{K}$ are inferred from the full dataset, and archetypes ${{z}^{\left(t\right)}}_{1},\ldots ,{{z}^{\left(t\right)}}_{K}$ are recomputed across $t\in 1,\ldots ,T$ bootstrap resamples. Because archetype labels are not identifiable across runs, bootstrap archetypes are aligned to the reference solution using Hungarian matching to solve the linear assignment problem

$${{\rm{\sigma }}}^{\left({\rm{t}}\right)}={{\rm{argmin}}}_{{\rm{\sigma }}\in {{\rm{S}}}_{{\rm{K}}}}\mathop{\sum }\limits_{{\rm{k}}=1}^{{\rm{K}}}{{||}{{\rm{z}}}_{k}-{{\rm{z}}}_{{\rm{\sigma }}\left({\rm{k}}\right)}^{\left({\rm{t}}\right)}{||}}_{2},$$

where ${S}_{K}$ denotes the set of all permutations of 1,…,K. For each archetype *k*, stability is quantified by the bootstrap variance

$${{\rm{v}}}_{{\rm{k}}}=\frac{1}{{\rm{TD}}}\mathop{\sum }\limits_{{\rm{t}}=1}^{{\rm{T}}}{{||}{{\rm{z}}}_{{\rm{k}}}^{\left({\rm{t}}\right)}-\bar{{{\rm{z}}}_{{\rm{k}}}}{||}}_{2}^{2},$$

with $\bar{{z}_{k}}=\frac{1}{T}{\sum }_{t=1}^{T}{z}_{k}^{\left(t\right)}$. Low bootstrap variance indicates stable, well-defined archetypes, whereas high variance suggests sensitivity to resampling and potential over-parameterization.

In practice, *K* is selected by jointly considering these criteria, favoring solutions that achieve high variance explained, low information-theoretic criterion, and stable archetypes under bootstrap resampling.

### Archetype characterization

To characterize each inferred archetype, we assign cells to archetypes using a smooth distance-based weighting defined in the same low-dimensional space used for archetypal analysis (typically PCA or Harmony-integrated principal components). For archetype *k* and cell *n*, we define the weight as

$${w}_{{kn}}=\frac{\exp \left(-\frac{{{||}{z}_{k}-{x}_{n}{||}}_{2}^{2}}{2{l}^{2}}\right)}{\mathop{\sum }\nolimits_{{n}^{{\prime} }=1}^{N}\exp \left(-\frac{{{||}{z}_{k}-{x}_{{n}^{{\prime} }}{||}}_{2}^{2}}{2{l}^{2}}\right)}$$

Here, ${z}_{k}$ denotes the position of archetype *k*, ${x}_{n}$ the position of cell *n*, and *l* is a length-scale parameter controlling how sharply weights decay with distance. This construction yields a soft assignment of cells to archetypes, ensures non-negativity of weights, and enforces $\mathop{\sum }\nolimits_{n}{w}_{{kn}}=1$ for each archetype. As a result, archetype characterization reflects continuous structure in the data rather than imposing discrete cell-state boundaries.

While the length scale *l* is a user-adjustable parameter, ParTIpy defaults to setting *l* as one-half of the median Euclidean distance between each archetype and the global mean of the data in the low-dimensional embedding

$$l=\frac{1}{2}{\rm{median}}\left(\left\{{{||}{z}_{k}-\bar{x}{||}}_{2},|,k=1,\ldots ,K\right\}\right)$$

where $\bar{x}=\frac{1}{N}{\sum }_{n=1}^{N}{x}_{n}$ denotes the global mean of the low-dimensional embedding.

This heuristic scales the kernel width to the overall spread of the archetype configuration, ensuring stable weighting across datasets with different intrinsic scales while avoiding manual parameter tuning.

Using the archetype weights, we compute archetype-specific feature profiles by aggregating cell-level measurements. For gene expression data, this aggregation is defined as

$${Y}^{\left(Z\right)}=W{Y}^{\left(X\right)},$$

where $W\in {R}^{K\times N}$ is the archetype weight matrix, ${Y}^{\left(X\right)}\in {R}^{N\times G}$ is the cell-by-gene expression matrix, and ${Y}^{\left(Z\right)}\in {R}^{K\times G}$ contains the aggregated expression profiles for each archetype. In practice, aggregation is performed on log1p-normalized gene expression values that are z-scored across cells prior to weighting. This highlights genes that vary across archetypes, while uniformly expressed genes yield z-scores close to zero.

The same weighting framework is used to associate continuous covariates (e.g., pseudotime, age, or quality-control metrics) with archetypes by replacing the expression matrix with the corresponding covariate vector.

For categorical covariates (e.g., disease status, sex, or donor identity), labels are one-hot encoded at the cell level, yielding a binary matrix $H\in \{{\mathrm{0,1}}\}^{N\times C}$. To account for unequal category sizes, each column is normalized to sum to one,

$${\widetilde{h}}_{{nc}}=\frac{{h}_{{nc}}}{\mathop{\sum }\nolimits_{{n}^{{\prime} }=1}^{N}{h}_{{n}^{{\prime} }c}},$$

and archetype–category enrichment scores are computed as

$$S=W\widetilde{H}\in {{\mathbb{R}}}_{+}^{K\times C}$$

While archetype characterization could in principle be based directly on the coefficient matrix *B* defining each archetype, this approach is impractical because *B* is typically extremely sparse by construction, limiting its ability to provide stable and interpretable feature aggregates. Similarly, archetype characterization could be based on the cell-level coefficients *A*, but these coefficients are not well-defined once the number of archetypes exceeds the embedding dimensionality plus one $K > \left(D+1\right)$.

### Functional enrichment analysis

To interpret archetype-specific transcriptional programs, we performed functional enrichment analysis on the archetype-aggregated expression profiles using decoupler-py (Badia-i-Mompel et al, 2022). By default, we applied the univariate linear model (ULM), which relates archetype expression profiles to predefined gene sets or regulons according to

$${y}_{k}^{\left(Z\right)}={\beta }_{0}+{\beta }_{1}w+\varepsilon .$$

Here, ${{y}_{k}}^{\left(Z\right)}$ denotes the expression profile of archetype *k* and *w* encodes gene-set or regulon weights. Intuitively, if genes with larger weights tend to be more highly expressed in archetype *k*, the estimated coefficient *β*_1_ will be positive and large in magnitude. For unweighted gene sets, such as MSigDB’s Hallmark gene sets (Liberzon et al, 2015; Subramanian et al, 2005), weights are binary and indicate gene-set membership. Enrichment scores are derived from the estimated effect *β*_1_, and statistical significance is assessed using the associated *t*-statistic. *P* values are corrected for multiple testing using the Benjamini–Hochberg procedure.

### Spatial mapping of archetypal programs

When spatial transcriptomics data from the same biological context are available, archetypal programs inferred from dissociated single-cell data can be projected onto spatial cells. We consider a dissociated dataset with log1p-normalized gene-expression matrix $Y\in {R}^{N\times G}$ and corresponding low-dimensional embedding $X\in {R}^{N\times D}$ from which archetypes are inferred. In the same biological context, we further assume the availability of a spatial single-cell dataset with log1p-normalized expression matrix ${Y}^{{\prime} }\in {R}^{{N}^{{\prime} }\times {G}^{{\prime} }}$, where typically ${G}^{{\prime} } < G$.

Because the dissociated dataset captures a larger number of genes and therefore provides a richer basis for archetype inference, archetypes are learned exclusively from the dissociated embedding *X*, yielding archetype-specific expression profiles ${Y}^{\left(Z\right)}\in {R}^{K\times G}$ as described above. To enable comparison with the spatial data, these archetype profiles are restricted to the intersection of genes shared between the dissociated and spatial datasets, yielding

$${Y}_{G}^{\left(Z\right)}\in {{\mathbb{R}}}^{K\times {G}^{{\prime} {\prime} }},$$

where ${G}^{{\prime} {\prime} }$ denotes the size of the gene intersection. Each spatial cell is then scored for association with each archetype by applying the univariate linear model (ULM) implemented in decoupler-py (Badia-i-Mompel et al, 2022) to the restricted archetype profiles. This yields, for every spatial cell and archetype, an enrichment score quantifying the degree to which the cell’s expression profile aligns with the corresponding archetypal program. These scores can be visualized across tissue space to generate spatial maps of archetypal program activity.

### Archetype crosstalk network inference

The observed distribution of cells across archetypes can be interpreted as a functional division of labor, where each archetype represents a specialization toward a distinct biological task. Ligand–receptor (LR) signaling is one potential mechanism that establishes and maintains this division of labor. To generate hypotheses about communication between archetypes, we infer archetype crosstalk networks by combining archetype-specific expression profiles with curated LR interaction databases. Crosstalk inference proceeds in three steps.

1. Identification of archetype-enriched genes.
  For each archetype *k* and gene *g*, we compute a specificity score
  $${s}_{{kg}}={y}_{{kg}}^{\left(Z\right)}-\mathop{\max }\limits_{{k}^{{\prime} }\ne k}{y}_{{k}^{{\prime} }g}^{\left(Z\right)}$$
  where ${Y}^{\left(Z\right)}\in {R}^{K\times G}$ denotes the matrix of archetype-specific expression profiles computed from z-scored, log1p-normalized single-cell expression. Genes with ${s}_{{kg}} > \tau$ (default τ=0.1) are considered enriched in archetype *k*.
2. Filtering to archetype-specific ligand–receptor pairs.
  Let *D* denote a curated set of cognate ligand–receptor pairs. For each ordered pair of archetypes $\left(k,{k}^{{\prime} }\right)$, we retain only those LR pairs whose ligand *l* is enriched in archetype *k* and whose receptor *r* is enriched in archetype ${k}^{{\prime} }$,
  $${{\mathscr{D}}}_{k\to {k}^{{\prime} }}=\left\{\left(l,r\right){\mathscr{\in }}\,{\mathscr{D}}\, \vert\,{l}\,{\rm{enriched}}\,{\rm{in}}\,k,r\,{\rm{enriched}}\,{\rm{in}}\,{k}^{{\prime} }\right\}$$
3. Construction of the crosstalk network.

For each ordered archetype pair $\left(k,{k}^{{\prime} }\right)$ with at least one LR connection, we introduce a directed edge $k\to {k}^{{\prime} }$. The edge weight is defined as

$${w}_{k\to {k}^{{\prime} }}=|{{\mathscr{D}}}_{k\to {k}^{{\prime} }}|,$$

corresponding to the number of supporting ligand–receptor interactions. The resulting weighted, directed graph summarizes potential signaling axes that may coordinate the division of labor among archetypes.

### Quantile-based enrichment analysis

As a complementary enrichment strategy aligned with prior ParTI studies (Korem et al, 2015; Adler et al, 2019; Hart et al, 2015), we implement a distance-ranking–based enrichment framework that directly links feature specificity to proximity to archetypes. This approach follows the original ParTI reference implementation.

For a fixed archetype *k*, we compute the Euclidean distance between the archetype position ${z}_{k}$ and the low-dimensional representation ${x}_{n}$ of each cell *n*,

$${d}_{{kn}}={{||}{z}_{k}-{x}_{n}{||}}_{2}.$$

Cells are ordered by increasing distance to the archetype, and distances are converted to ranks to ensure robustness to absolute distance scale.

Ranked cells are partitioned into disjoint quantile bins of approximately equal size, with each bin containing a fixed fraction of the cells (default: 10%). Larger datasets permit finer stratification while maintaining sufficiently large bin sizes to ensure statistical robustness. Enrichment is evaluated by contrasting a foreground set of cells closest to the archetype with a background set consisting of all remaining cells, although alternative contrasts such as closest versus furthest bins are also supported.

For continuous features such as gene expression, enrichment is assessed by contrasting foreground and background cells using a Mann–Whitney *U*-test by default, with the option to apply Welch’s two-sample *t*-test. Multiple testing across features and archetypes is controlled using the Benjamini–Hochberg procedure. As an additional specificity criterion, features can be required to attain maximal mean or median expression in the bin closest to the archetype. This requirement is most appropriate for continuous covariates such as age or pseudotime, but may be unstable for sparse features such as gene expression with a high fraction of zero values.

For categorical features (e.g., disease status or sex), each category level is treated as a one-versus-rest indicator variable. Enrichment is evaluated by comparing category frequencies across quantile bins, defining an enrichment curve as the ratio between the category frequency within each bin and its global frequency across all cells. Categories are required to show maximal enrichment in the bin closest to the archetype. Statistical significance of over-representation in the foreground relative to the background is assessed using a hypergeometric test, with multiple testing correction performed using the Benjamini–Hochberg procedure.

### Hepatocyte analysis

We analyzed hepatocyte scRNA-seq data (Ben-Moshe et al, 2022) following the workflow summarized in section “Using ParTIpy to characterize cellular task allocation”. Expression preprocessing consisted of total-count normalization (sc.pp.normalize_total) and log1p transformation (sc.pp.log1p), followed by highly variable gene (HVG) selection (sc.pp.highly_variable_genes) and principal component analysis (PCA) computed on the selected HVGs (sc.pp.pca with mask_var =  “highly_variable”). In addition to the normalized log-expression, we constructed a z-scored expression representation by scaling each gene across cells with clipping at a maximum absolute value of 10 (sc.pp.scale with max_value = 10). This z-scored representation was used for archetype characterization and downstream enrichment analyses (pt.compute_archetype_expression with layer =  “z_scaled”).

To determine the dimensionality of the archetypal-analysis input space, we performed shuffled PCA diagnostics (pt.compute_shuffled_pca with mask_var =  “highly_variable”) and retained the first D = 3 principal components whose variance exceeded the shuffled baseline. We then evaluated the number of archetypes using complementary selection criteria across $K\in 2,\ldots ,8$ (pt.compute_selection_metrics) and assessed robustness using bootstrap-based archetype stability analysis with 50 resamples (pt.compute_bootstrap_variance). Based on joint consideration of reconstruction quality, information-theoretic criterion, and bootstrap stability, we selected a final model with K=4 archetypes.

Archetype characterization was performed using the distance-based kernel weighting scheme described in section “Archetype characterization”, with automatic selection of the kernel length scale (pt.compute_archetype_weights). Using these weights, we computed archetype-aggregated expression profiles from the z-scored expression matrix, yielding one characteristic transcriptional profile per archetype (pt.compute_archetype_expression with layer = “z_scaled”).

To interpret archetypal programs, we performed pathway enrichment analysis using Reactome (Gillespie et al, 2022) gene sets via the univariate linear model (ULM) implemented in decoupler-py (dc.mt.ulm) (Badia-i-Mompel et al, 2022) (section “Functional enrichment analysis”). Reactome gene sets were obtained from MSigDB (dc.op.resource with resource = “MSigDB”) and restricted to the reactome_pathways collection. To improve interpretability and biological specificity, we removed overly generic pathways and context-irrelevant terms, including broad regulatory processes (e.g., RESPONSE_TO, GENE_EXPRESSION, and STIMULATED_TRANSCRIPTION) and virus-related pathways (e.g., SARS_COV). Pathways were further filtered by size, retaining only gene sets containing between 5 and 80 genes. Pathway activities were inferred on the archetype-aggregated expression profiles, and enriched pathways were retained after multiple-testing correction using the Benjamini–Hochberg procedure at *p* ≤ 0.05.

To assess whether inferred archetypal programs correspond to spatially organized functional zonation, we mapped archetypal programs onto single-cell–resolved spatial transcriptomics data from the same biological context, following section “Spatial mapping of archetypal programs”. Spatial data were restricted to genes shared with the scRNA-seq–derived archetype programs. Spatial cells were filtered to retain profiles with sufficient total counts (total counts >200) and processed using total-count normalization (sc.pp.normalize_total) followed by log1p transformation (sc.pp.log1p). Archetypal program activity in spatial cells was quantified using the univariate linear model (ULM), with archetype-aggregated expression profiles used as the reference network (dc.mt.ulm). Resulting archetype scores were z-scored prior to visualization.

Finally, to generate hypotheses about potential signaling interactions underlying the inferred division of labor, we performed archetype crosstalk network inference as described in section “Archetype crosstalk network inference”. Archetype-specific genes were identified based on specificity scores (pt.get_specific_genes_per_archetype with min_score = 0.05), and ligands and receptors were obtained from curated ligand–receptor resources (li.rs.select_resource with resource = “mouseconsensus”).

### Fibroblast cross-condition analysis

Single-nucleus RNA-seq data from human dilated and hypertrophic cardiomyopathy, together with non-failing donor hearts, were obtained from (Chaffin et al, 2022) (see Table EV2). Cells were subset to fibroblast annotations (Fibroblast_I, Activated_fibroblast, Fibroblast_II). Low-quality cells and lowly expressed genes were filtered using sc.pp.filter_cells (min_genes = 100) and sc.pp.filter_genes (min_cells = 10). Disease labels were collapsed to non-failing (NF) and cardiomyopathy (CM).

Counts were total-count normalized (sc.pp.normalize_total) and log-transformed (sc.pp.log1p). Highly variable genes were selected (sc.pp.highly_variable_genes, default arguments), and principal component analysis (PCA) was computed on the selected HVGs (sc.pp.pca with mask_var = “highly_variable”, n_comps = 50). A z-scored expression layer was stored for downstream archetype characterization (sc.pp.scale with max_value = 10). To correct donor-specific effects, batch correction across donors was performed using Harmony (Korsunsky et al, 2019) (hm.run_harmony) on the PCA embedding, yielding a Harmony-integrated space (X_pca_harmony). Archetypal analysis was performed in this Harmony-corrected embedding using the first 16 components, with the effective dimensionality determined using a shuffled PCA baseline (pt.compute_shuffled_pca), and a K = 3 archetype model was fitted. For the shuffled PCA baseline, PCA was applied to the mean-centered gene-expression matrix (log1p-normalized highly variable genes). To estimate a null distribution, we generated 50 shuffled datasets by independently permuting each gene (column) across cells, thereby preserving marginal gene distributions while removing gene–gene covariance. PCA was fitted to each shuffled matrix, and components were retained if their explained variance in the original data exceeded the shuffled mean plus one standard deviation, resulting in 16 components used for downstream archetypal analysis.

Model selection followed the ParTIpy workflow. The number of archetypes was evaluated using variance explained and information-criterion curves (pt.compute_selection_metrics) together with bootstrap-based archetype stability analysis (pt.compute_bootstrap_variance). Archetype weights were computed using pt.compute_archetype_weights.

For cross-condition comparisons, donor-level pseudobulks were computed by averaging Harmony coordinates across fibroblasts within each donor. Donor pseudobulks were projected into archetype coefficient space (pt.AA(…).transform), and Euclidean distances to each archetype were computed. Disease effects on donor-level distances were assessed using two-sided Welch’s *t*-tests for each archetype.

Archetype expression profiles were computed for raw counts, log1p-normalized values, and z-scored values (pt.compute_archetype_expression), restricting to genes expressed in more than 100 cells. For donor-level summaries of proximity to archetype 2, cells were assigned to their maximum-weight archetype using the coefficient matrix A, and the number of NF cells closest to archetype 2 was tabulated per donor.

Transcription factor (TF) activity was inferred from archetype z-scored expression profiles using decoupler’s univariate linear model (ULM) (Badia-i-Mompel et al, 2022). CollecTRI regulons (Müller-Dott et al, 2023) were retrieved (dc.op.collectri with organism = “human”) and scored (dc.mt.ulm). Pathway activity was inferred analogously using PROGENy (dc.op.progeny with organism = “human”, followed by dc.mt.ulm). NABA Matrisome (Naba *et al*, 2016) gene sets were extracted from the MSigDB resource (dc.op.resource with resource = “MSigDB”), excluding cancer-specific subsets, and scored using the same ULM workflow. For all ULM-based analyses, two-sided *p* values were adjusted using the Benjamini–Hochberg procedure.

## Supplementary information

Peer Review File

Appendix

Table EV1

Table EV2

Table EV3

Source data Fig. 2

Source data Fig. 3

Source data Fig. 4

Figure EV1 Source Data

Figure EV2 Source Data

Expanded View Figures

## Data Availability

For the datasets used in the benchmarking part, see Table EV1. For the datasets used to compare the ParTI MATLAB package and ParTIpy, see Table EV2. For the datasets used in the scalability and time benchmark, see Table EV3. Scripts to download the data and reproduce the analyses described in the manuscript are available at https://github.com/saezlab/ParTIpy_paper. All analyses in this manuscript were conducted using ParTIpy v0.2.0. ParTIpy is available from PyPI (https://pypi.org/project/partipy). The source code is available on GitHub https://github.com/saezlab/ParTIpy. Documentation is available at https://partipy.readthedocs.io. The source data of this paper are collected in the following database record: biostudies:S-SCDT-10_1038-S44320-026-00209-6.
