# Supplementary material for: ParTIpy: a scalable framework for archetypal analysis and Pareto task inference
Source: Mol Syst Biol. 2026 Jun 25;22(8):1252–69. doi: 10.1038/s44320-026-00209-6 (PMC13433804; doi:10.1038/s44320-026-00209-6)
Supplement: Supplementary file 1 [file 44320_2026_209_MOESM11_ESM.pdf]

Expanded View Figures

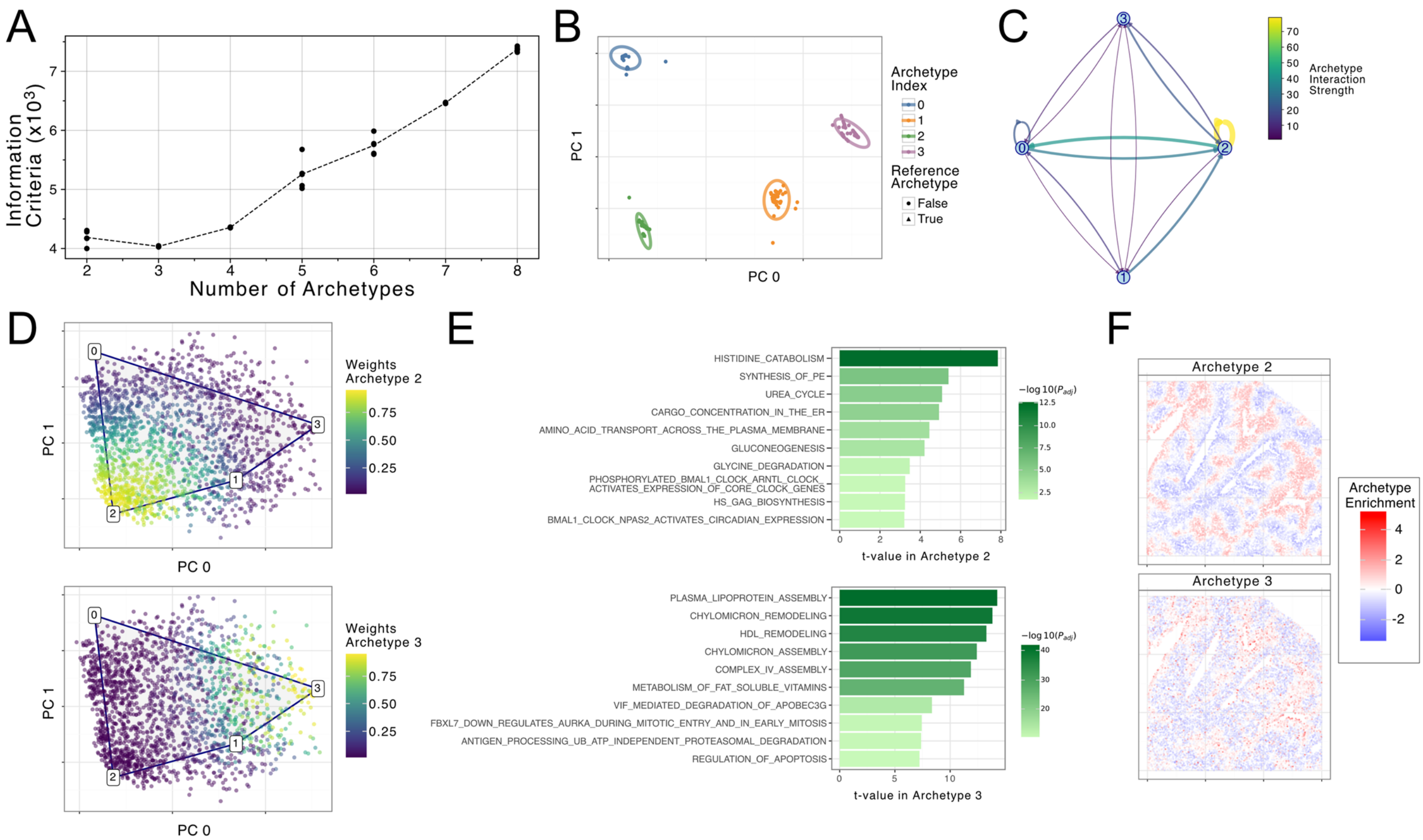

Figure EV1. Example analysis on hepatocyte data from (Ben-Moshe et al, 2022).

(**A**) Information criterion for archetypal analysis plotted for two to eight archetypes. (**B**) Two-dimensional PC plot showing the archetypal positions obtained from performing the optimization on 30 different bootstrap samples. (**C**) Crosstalk analysis shows strong self-interaction in archetype 2, and strong interaction between archetypes 0 and 2. (**D**) Two-dimensional PC plots showing the weights used to characterize archetypes 2 and 3. (**E**) Barplots showing the top enriched Reactome pathways for archetypes 2 and 3. *t* values and *P* values are based on decoupler ULM run on archetype-aggregated (z-scored) expression profiles, reporting two-sided p-values from the t-statistic of the gene-set coefficient and BH-adjusted across Reactome pathways. (**F**) Spatial mapping of archetypes 2 and 3. Source data are available online for this figure.

 © The Author(s)

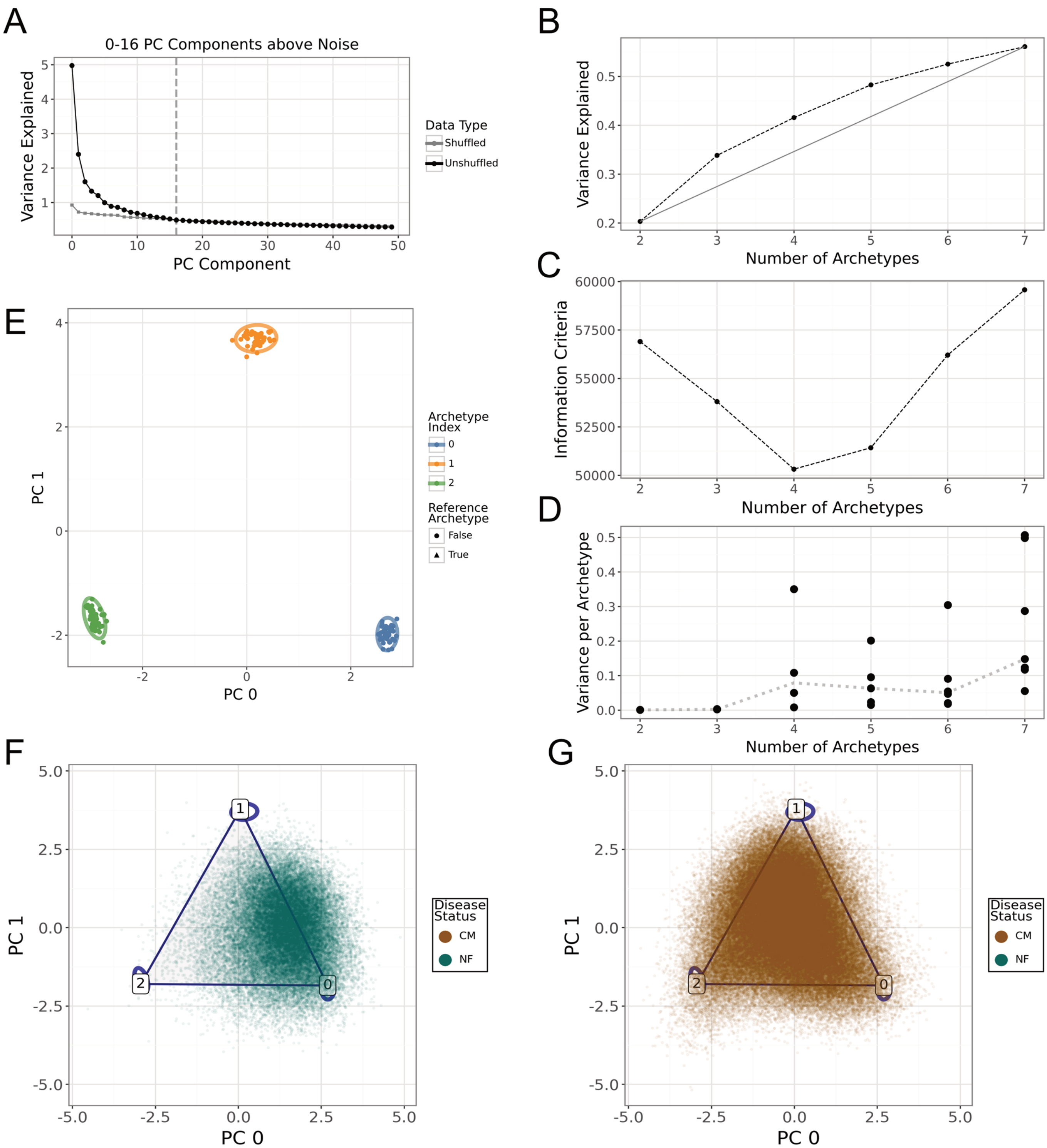
A
0-16 PC Components above Noise
Variance Explained
PC Component
Data Type
Shuffled
Unshuffled
B
Variance Explained
Number of Archetypes
C
Information Criteria
Number of Archetypes
D
Variance per Archetype
Number of Archetypes
E
PC 1
PC 0
Archetype Index
0
1
2
Reference Archetype
False
True
F
PC 1
PC 0
Disease Status
CM
NF
G
PC 1
PC 0
Disease Status
CM
NF

Figure EV2. Model selection and robustness diagnostics for the fibroblast cross-condition analysis.

(**A**) Shuffled principal component (PC) analysis used to determine the dimensionality of the input space for archetypal analysis. Variance explained by PCs from the original data (black) is compared to shuffled data (gray); PCs exceeding the shuffled baseline (PCs 0–16) were retained for downstream analysis. (**B**) Variance explained by archetypal analysis as a function of the number of archetypes (K = 2–7). (**C**) Information criterion for archetypal analysis evaluated across archetype numbers. (**D**) Bootstrap-based archetype stability analysis. Points show positional variance of individual archetypes across bootstrap replicates for each K; the dashed line indicates the mean variance across archetypes. Lower variance corresponds to more stable archetype solutions. (**E**) Two-dimensional PC projection showing the locations of archetypes inferred across bootstrap replicates for K = 3. Ellipses indicate the dispersion of bootstrap archetype positions around the reference archetypes, illustrating high positional stability for K = 3. (**F**, **G**) Two-dimensional PC projection of single-cell fibroblast profiles colored by disease status (NF and CM), with the inferred three-archetype simplex overlaid. Both conditions populate the same archetypal geometry, while disease-associated shifts are evident as changes in cell density toward specific archetypal programs. Source data are available online for this figure.

 © The Author(s)
