# Supplementary material for: ParTIpy: a scalable framework for archetypal analysis and Pareto task inference
Source: Mol Syst Biol. 2026 Jun 25;22(8):1252–69. doi: 10.1038/s44320-026-00209-6 (PMC13433804; doi:10.1038/s44320-026-00209-6)
Supplement: Supplementary file 2 [file 44320_2026_209_MOESM1_ESM.pdf]

ParTIpy: A Scalable Framework for Archetypal Analysis and Pareto Task Inference

Philipp Schäfer, Leoni Zimmermann, Paul Burmedi, Avia Walfisch, Noa Goldenberg, Shira Yonassi, Einat Tamar, Miri Adler, Jovan Tanevski, Ricardo Ramirez Flores, and Julio Saez-Rodriguez

Corresponding author(s): Ricardo Ramirez Flores (flores@ebi.ac.uk)

Review Timeline:

Submission Date:	9th Dec 25
Editorial Decision:	10th Dec 25
Revision Received:	4th Mar 26
Editorial Decision:	25th Mar 26
Revision Received:	30th Mar 26
Accepted:	2nd Apr 26

Editor: Jingyi Hou

Transaction Report: This manuscript was transferred to Molecular Systems Biology following peer review at Review Commons.

Review #1

1. Evidence, reproducibility and clarity:

Evidence, reproducibility and clarity (Required)

Summary

The paper "ParTIpy: A Scalable Framework for Archetypal Analysis and Pareto Task Inference" presents ParTIpy, an open-source Python package that modernizes and scales the Pareto Task Inference (ParTI) framework for analyzing biological trade-offs and functional specialization. Unlike the earlier MATLAB implementation, which required a commercial license and was limited in scalability, ParTIpy leverages Python's open ecosystem and integration with tools such as scverse to make archetypal analysis more accessible, flexible, and compatible with modern biological data workflows. Through advanced optimization and coreset algorithms, it efficiently handles large scale single cell and spatial transcriptomics datasets. ParTIpy identifies "archetypes", or optimal phenotypic extremes, to reveal how cells balance competing functional programs. The paper demonstrates its application in modeling hepatocyte specialization across the liver lobule, highlighting spatial patterns of metabolic division of labor.
Overall, ParTIpy represents a modern, accessible, and scalable Python-based solution for exploring biological trade-offs and resource allocation in high-dimensional data. The paper is clearly written and addresses an important methodological gap. However, the enrichment analysis differs from the original ParTI framework and should be discussed more explicitly, and the documentation and tutorials, while helpful, could be refined to improve usability and reproducibility.

Major Comments

1. The archetype enrichment analysis used in this paper differs from the original enrichment analysis implemented in ParTI. This is acceptable, but:

a. The authors should explicitly state and discuss the differences between the two approaches.

b. The enrichment analysis should be made more systematic. For each tested feature (e.g. gene or pathway), the analysis should report a p-value for the hypothesis that the feature is enriched near an archetype - that is, its expression (or value) is high close to the archetype

and decreases with distance. Appropriate multiple-hypothesis correction should also be applied.
2. The package documentation on GitHub and ReadTheDocs is a major strength, but the tutorials can be improved for clarity and accessibility:

a. The documentation should list external dependencies that need to be installed seperately, e.g. pybiomart.

b. The dataset used in the Quickstart demo appears to be inaccessible or extremely slow to download (the function load_hepatocyte_data_2() did not complete even after 30 minutes, at least in my experience). The authors should verify data availability on Zenodo and consider providing a smaller or cached version to make the demo more reliable and reproducible.

c. The tutorial order could be more intuitive - for instance, "archetype crosstalk network" appears before "archetypal analysis". Consider starting with the simulated dataset and presenting the full pipeline before moving to more complex real-world examples.

Minor comments

1. In the Python function, the parameter "optim" could use more descriptive option names - for example, renaming "projected_gradients" to "PCHA" would make it clearer and more consistent with terminology used in the paper.
2. In the Quickstart preprocessing, the authors use the following code:
sc.pp.normalize_total(adata)
sc.pp.log1p(adata)
However, they do not specify the target sum in the normalize_total function. The authors should ensure that the data values before the logarithmic transformation span several orders of magnitude (e.g., 0-10,000); if normalization is performed to a sum of 1, the log transformation becomes ineffective.

Referee cross-commenting

I agree with Reviewer #2 observation that the paper's contribution is primarily technical; however, I consider this technical advance to be an important and timely one that will enable many biologists to apply archetypal analysis more effectively in their own work.

2. Significance:

Significance (Required)

This study presents ParTIpy, a Python-based implementation of Pareto Task Inference (ParTI) that makes archetypal analysis more accessible, scalable, and compatible with modern single-cell and spatial transcriptomics workflows. Its main strength lies in translating a conceptually powerful but technically limited MATLAB framework into an open-source, efficient Python package, enabling wider use in computational biology. The package is well-documented, which further enhances its accessibility and adoption potential, though documentation could be improved to enhance reproducibility and ease of use. It will be of interest to computational systems biologists, particularly those working with omics data, and those interested in studying functional trade-offs and resource allocation.

Review #2

1. Evidence, reproducibility and clarity:

Evidence, reproducibility and clarity (Required)

Summary

This paper introduces the software ParTIpy, a scalable Python implementation of Pareto Task Inference (ParTI), designed to infer functional trade-offs in biological systems through archetypal analysis. The framework modernizes the previous toolbox with efficient optimization, memory-saving coreset construction, and integration with the scverse ecosystem for single-cell transcriptomic data.

Using hepatocytes scRNA-seq data as a test case, the authors identify archetypes corresponding to distinct gene expression patterns. These archetypes align with known liver domains in spatial transcriptomics data, validating both the method's interpretability and its biological relevance.

Major comments

(1) Conclusions

The core computational and biological claims are well supported. ParTIpy clearly scales better than earlier implementations and reproduces known biological structure. However, claims about "scalability to large datasets" should be further qualified (see below).

(2) Claims

Archetypal analysis based on current matrix computation formulation is non-parametric, and new data require recomputation of archetypes. Therefore, the method cannot generalize to unseen data in the way deep learning approaches, which could be further acknowledged and clarified.

(3) Additional suggested analyses or experiments

1. Absolute performance benchmarks : it's suggested to report wall-clock time and memory for a few dataset sizes (10k, 100k, 1M cells).
2. Coreset sensitivity analysis: Could authors show how coreset size affects archetype positions and biological interpretation?

Referee cross-commenting

I agree with the other reviewer's suggestion to check consistency and reproducibility with previous implementation, and enhance the tutorial of the software for users from a biological background. Combined with my comments to further improve the biological application showcase, the revised manuscript could be an impactful contribution to the field, if these comments could be properly addressed.

2. Significance:

Significance (Required)

(1) Advance

This paper is primarily a technical contribution. It modernizes the Pareto Task Inference framework into a scalable and user-friendly Python implementation, which is valuable. However, to further improve its significance especially for the broader biological audience, more detailed analysis could be performed (see below)

(2) Biological scope and applications [optional]

The current biological validation in hepatocyte is technically fine but limited in breadth and impact. It demonstrates that ParTIpy works but falls in short of showing what new insights it can reveal. Several promising applications could be further explored:

1) Cross-condition comparisons: could ParTIpy quantify how the Pareto front shifts between conditions (e.g., normal vs. tumor, treated vs. control)?

2) Transient or plastic states: Cells with mixed archetype weights or high mixture entropy can be interpreted as transient, functionally flexible states. ParTIpy can quantify such transience geometrically, even in static data, which providing a competitive counterpart to models like CellRank or CellSimplex (https://doi.org/10.1093/bioinformatics/btaf119).

Because the manuscript does not explore these scenarios, the biological impact remains narrow, and the framework's broader interpretive power is somehow underrepresented.

(3) Audience and impact

The paper will interest computational biologists, systems biologists, and bioinformaticians focused on single-cell analysis, and its impact will grow substantially if the authors demonstrate more biological applications.

(4) Reviewer expertise
Computational biology, single-cell transcriptomics, machine learning, computational math

Revision Plan

Manuscript number: RC-2025-03221
Corresponding author(s): Ricardo, Ramirez Flores

[The "revision plan" should delineate the revisions that authors intend to carry out in response to the points raised by the referees. It also provides the authors with the opportunity to explain their view of the paper and of the referee reports.

The document is important for the editors of affiliate journals when they make a first decision on the transferred manuscript. It will also be useful to readers of the reprint and help them to obtain a balanced view of the paper.

If you wish to submit a full revision, please use our "Full Revision" template. ***It is important to use the appropriate template to clearly inform the editors of your intentions.****]*

1. General Statements [optional]

This section is optional. Insert here any general statements you wish to make about the goal of the study or about the reviews.

Thank you for providing an assessment of our manuscript. We suggest here a revision plan to address the points raised by the reviewers regarding code documentation, benchmarking, and biological applications.

As part of the revisions implemented we have:

- Clarified the management of dependencies of our package
- Fixed the data download run times of test data
- Clarified the parameters of the normalization and optimization functions

We plan to:

- Extend our manuscript to include a section on cross-condition analysis that builds on our tutorials, where we will illustrate how ParTIpy can quantify shifts in the distribution of fibroblasts across the functional space defined by archetypal analysis between healthy and failing hearts.
- Extend our benchmarks of scalability of coresets, by reporting wall-clock time and peak memory usage across distinct data sizes.
- Extend our benchmarks of stability of coresets, by reporting the similarity of the estimated archetypes based on the original versus the sampled data.
- Include the original enrichment analysis of ParTI to provide users with distinct options to work with the archetypes, and provide a larger discussion on the distinct strategies.

We believe these revisions will strengthen our **software manuscript** and will help us to provide a robust and practical tool to analyze functional trade-offs from biological data. Our responses to individual comments are below in **blue**.

2. Description of the planned revisions

Insert here a point-by-point reply that explains what revisions, additional experimentations and analyses are planned to address the points raised by the referees.

Reviewer #1

Summary
The paper "ParTIpy: A Scalable Framework for Archetypal Analysis and Pareto Task Inference" presents ParTIpy, an open-source Python package that modernizes and scales the Pareto Task Inference (ParTI) framework for analyzing biological trade-offs and functional specialization. Unlike the earlier MATLAB implementation, which required a commercial license and was limited in scalability, ParTIpy leverages Python's open ecosystem and integration with tools such as scverse to make archetypal analysis more accessible, flexible, and compatible with modern biological data workflows. Through advanced optimization and coreset algorithms, it efficiently handles large scale single cell and spatial transcriptomics datasets. ParTIpy identifies "archetypes", or optimal phenotypic extremes, to reveal how cells balance competing functional programs. The paper demonstrates its application in modeling hepatocyte specialization across the liver lobule, highlighting spatial patterns of metabolic division of labor.
Overall, ParTIpy represents a modern, accessible, and scalable Python-based solution for exploring biological trade-offs and resource allocation in high-dimensional data. The paper is clearly written and addresses an important methodological gap. However, the enrichment analysis differs from the original ParTI framework and should be discussed more explicitly, and the documentation and tutorials, while helpful, could be refined to improve usability and reproducibility.

Major Comments

1. The archetype enrichment analysis used in this paper differs from the original enrichment analysis implemented in ParTI. This is acceptable, but:
a. The authors should explicitly state and discuss the differences between the two approaches.
b. The enrichment analysis should be made more systematic. For each tested feature (e.g. gene or pathway), the analysis should report a p-value for the hypothesis that the feature is enriched near an archetype - that is, its expression (or value) is high close to the archetype and decreases with distance. Appropriate multiple-hypothesis correction should also be applied.

We thank the reviewer for this valuable comment and agree that the differences between our enrichment analysis and the original ParTI implementation should be stated more explicitly. We will incorporate the original enrichment algorithm into ParTIpy, enabling users to select their preferred method. In the revised manuscript, we will note that two enrichment algorithms are available and describe both in greater detail in the supplementary methods section. We also note that the current enrichment analysis already reports p-values adjusted for multiple hypothesis testing.

Reviewer #2

Summary

This paper introduces the software ParTIpy, a scalable Python implementation of Pareto Task Inference (ParTI), designed to infer functional trade-offs in biological systems through archetypal analysis. The framework modernizes the previous toolbox with efficient optimization, memory-saving coreset construction, and integration with the scverse ecosystem for single-cell transcriptomic data.

Using hepatocytes scRNA-seq data as a test case, the authors identify archetypes corresponding to distinct gene expression patterns. These archetypes align with known liver domains in spatial transcriptomics data, validating both the method's interpretability and its biological relevance.

Major comments

(1) Conclusions
The core computational and biological claims are well supported. ParTIpy clearly scales better than earlier implementations and reproduces known biological structure. However, claims about "scalability to large datasets" should be further qualified (see below).

We will implement further performance benchmarks as discussed below.

(2) Claims
Archetypal analysis based on current matrix computation formulation is non-parametric, and new data require recomputation of archetypes. Therefore, the method cannot generalize to unseen data in the way deep learning approaches, which could be further acknowledged and clarified.

We thank the reviewer for this insightful comment. We agree that deep learning frameworks are typically amortized, allowing them to generalize to unseen data without retraining, and we will clarify this distinction in the discussion of the revised manuscript. However, we note that mapping new cells into an existing archetypal space is computationally inexpensive, as it only requires solving a single convex optimization problem.

(3) Additional suggested analyses or experiments

1）Absolute performance benchmarks : it's suggested to report wall-clock time and memory for a few dataset sizes (10k, 100k, 1M cells).

We thank the reviewer for this helpful suggestion. We will extend our existing performance benchmarks to (a) include larger datasets (~1 million cells) and (b) report wall-clock time and peak memory usage, providing a clearer assessment of ParTIpy’s scalability.

2）Coreset sensitivity analysis: Could authors show how coreset size affects archetype positions and biological interpretation?

We thank the reviewer for this helpful suggestion. We will extend the coreset benchmark to quantify how coreset size affects both archetype positions and biological interpretation. Specifically, we will match archetypes across coreset sizes by solving the linear sum assignment problem, as we currently do when comparing bootstrap samples. We will then compare the distances between archetypes inferred from the full dataset and those obtained from different coreset sizes. In addition to measuring displacement, we will assess biological stability by comparing the gene expression vectors of corresponding archetypes as well as their enriched pathways (using metrics such as cosine similarity and Jaccard index).

Referee cross-commenting

I agree with the other reviewer's suggestion to check consistency and reproducibility with previous implementation, and enhance the tutorial of the software for users from a biological background. Combined with my comments to further improve the biological application showcase, the revised manuscript could be an impactful contribution to the field, if these comments could be properly addressed.

(1) Advance

This paper is primarily a technical contribution. It modernizes the Pareto Task Inference framework into a scalable and user-friendly Python implementation, which is valuable. However, to further improve its significance especially for the broader biological audience, more detailed analysis could be performed (see below)

(2) Biological scope and applications [optional]

The current biological validation in hepatocyte is technically fine but limited in breadth and impact. It demonstrates that ParTIpy works but falls in short of showing what new insights it can reveal. Several promising applications could be further explored:

1) Cross-condition comparisons: could ParTIpy quantify how the Pareto front shifts between conditions (e.g., normal vs. tumor, treated vs. control)?

We thank the reviewer for this valuable suggestion. We have shown ParTIpy’s applicability to cross-condition settings in our online tutorials (https://partipy.readthedocs.io/en/latest/notebooks/cross_condition_lupus.html). However, we agree that a more explicit mention in the manuscript is needed. Thus, we will include a cross-condition analysis as a second application in the revised manuscript, focusing on

fibroblasts from heart failure patients from Amrute, et. al. (2023) [1]. This will illustrate how ParTIpy can quantify shifts in the distribution of cells across the functional space defined by archetypal analysis.

Because the manuscript does not explore these scenarios, the biological impact remains narrow, and the framework's broader interpretive power is somehow underrepresented.

We hope that the additional application included in the revised manuscript helps better illustrate the framework's strength. We would also like to note that the online tutorials provide a comprehensive overview of ParTIpy's functionality, as we expect these will serve as a primary entry point for many researchers interested in archetypal analysis and Pareto Task Inference.

(3) Audience and impact

The paper will interest computational biologists, systems biologists, and bioinformaticians focused on single-cell analysis, and its impact will grow substantially if the authors demonstrate more biological applications.

(4) Reviewer expertise
Computational biology, single-cell transcriptomics, machine learning, computational math

3. Description of the revisions that have already been incorporated in the transferred manuscript

Please insert a point-by-point reply describing the revisions that were already carried out and included in the transferred manuscript. If no revisions have been carried out yet, please leave this section empty.

Reviewer #1

2. The package documentation on GitHub and ReadTheDocs is a major strength, but the tutorials can be improved for clarity and accessibility:

We thank the reviewer for this positive feedback. Indeed, providing comprehensive documentation to facilitate ease of adoption was a major motivation behind this project. In response to the reviewer's suggestions, we have revised the tutorials to further improve their clarity, structure, and accessibility, as detailed below.

a. The documentation should list external dependencies that need to be installed seperately, e.g. pybiomart.

We thank the reviewer for pointing this out. We had added all dependencies under the optional-dependencies.extra header, which allows users to run `pip install partipy[extra]` to be able to run all tutorial notebooks. However, we forgot to explain that in the tutorial or Readme page, which we corrected now. The Readme now reads:

```
Install the latest stable full release from PyPI with the extra
dependencies (e.g., `pybiomart`, `squidpy`, `liana`) that are
required to run every tutorial:

```
pip install partipy[extra]
```
```

Additionally we include clarifications in every tutorial notebook that uses additional dependencies: "To run this notebook, install ParTIpy with the tutorial extras: `pip install partipy[extra]`".

b. The dataset used in the Quickstart demo appears to be inaccessible or extremely slow to download (the function load_hepatocyte_data_2() did not complete even after 30 minutes, at least in my experience). The authors should verify data availability on Zenodo and consider providing a smaller or cached version to make the demo more reliable and reproducible.

We thank the reviewer for this helpful comment. We agree that the previous implementation of load_hepatocyte_data_2() was not reliable due to slow download speeds from Zenodo. To address this, we now host the required AnnData object on figshare (https://figshare.com/articles/dataset/scRNA-seq_hepatocyte_data_from_Ben-Moshe_et_al_2022_/30588713?file=59459459), ensuring faster and more stable access for the Quickstart tutorial via `scanpy.read`:

```
adata = sc.read("data/hepatocyte_processed.h5ad",
backup_url="https://figshare.com/ndownloader/files/59459459")
adata
```

c. The tutorial order could be more intuitive - for instance, "archetype crosstalk network" appears before "archetypal analysis". Consider starting with the simulated dataset and presenting the full pipeline before moving to more complex real-world examples.

We thank the reviewer for this helpful suggestion and agree that the previous ordering was not intuitive. We have reordered the tutorials such that the notebook introducing archetypal analysis now appears first, followed by the Quickstart tutorial and the subsequent applied examples.

Minor comments

1. In the Python function, the parameter "optim" could use more descriptive option names - for example, renaming "projected_gradients" to "PCHA" would make it clearer and more consistent with terminology used in the paper.

We thank the reviewer for this helpful suggestion. We agree that the previous naming could be misleading. While PCHA does not precisely describe the underlying algorithm, it is the term most users are familiar with from the literature. We have therefore updated the function to accept both "PCHA" and "projected_gradients", which now map to the same underlying optimization routine.

2. In the Quickstart preprocessing, the authors use the following code:
sc.pp.normalize_total(adata)
sc.pp.log1p(adata)
However, they do not specify the target sum in the normalize_total function. The authors should ensure that the data values before the logarithmic transformation span several orders of magnitude (e.g., 0-10,000); if normalization is performed to a sum of 1, the log transformation becomes ineffective.

We thank the reviewer for this helpful comment. By default, sc.pp.normalize_total scales the counts in each cell to the median total counts across all cells, which preserves the typical range of expression values prior to logarithmic transformation. We therefore consider this default

behavior appropriate for the Quickstart example. Nonetheless, we will clarify this explicitly in the tutorial to avoid confusion.

Referee cross-commenting

I agree with Reviewer #2 observation that the paper's contribution is primarily technical; however, I consider this technical advance to be an important and timely one that will enable many biologists to apply archetypal analysis more effectively in their own work.

We thank the reviewer for this positive and encouraging assessment.

Reviewer #1 (Significance (Required)):

This study presents ParTIpy, a Python-based implementation of Pareto Task Inference (ParTI) that makes archetypal analysis more accessible, scalable, and compatible with modern single-cell and spatial transcriptomics workflows. Its main strength lies in translating a conceptually powerful but technically limited MATLAB framework into an open-source, efficient Python package, enabling wider use in computational biology. The package is well-documented, which further enhances its accessibility and adoption potential, though documentation could be improved to enhance reproducibility and ease of use. It will be of interest to computational systems biologists, particularly those working with omics data, and those interested in studying functional trade-offs and resource allocation.

We appreciate the reviewer's positive evaluation and are encouraged by their recognition of ParTIpy's relevance and potential impact in computational biology.

4. Description of analyses that authors prefer not to carry out

Please include a point-by-point response explaining why some of the requested data or additional analyses might not be necessary or cannot be provided within the scope of a revision. This can be due to time or resource limitations or in case of disagreement about the necessity of such additional data given the scope of the study. Please leave empty if not applicable.

Reviewer #2

The current biological validation in hepatocyte is technically fine but limited in breadth and impact. It demonstrates that ParTIpy works but falls in short of showing what new insights it can reveal. Several promising applications could be further explored:

2) Transient or plastic states: Cells with mixed archetype weights or high mixture entropy can be interpreted as transient, functionally flexible states. ParTIpy can quantify such transience geometrically, even in static data, which providing a competitive counterpart to models like CellRank or CellSimplex (https://doi.org/10.1093/bioinformatics/btaf119).

We thank the reviewer for this interesting suggestion. While we agree that quantifying transient or plastic states based on archetype mixtures is an intriguing idea, validating whether cells with mixed archetype weights ("generalists") truly represent transient states would require additional data modalities such as temporal or lineage-tracing measurements. Although we find this direction highly interesting, given that the manuscript is intended as a software paper, we prefer to focus on more directly supported applications of cross-condition data, where labeled data is available.

However, we will expand our discussion to relate ParTIpy with CellSimplex since we believe this is an interesting angle that future users could explore.

5. References

1. Amrute, J. M. *et al.* Defining cardiac functional recovery in end-stage heart failure at single-cell resolution. *Nat. Cardiovasc. Res.* **2**, 399–416 (2023).

10th Dec 2025

Manuscript Number: MSB-2025-13511-T
Title: ParTIpy: A Scalable Framework for Archetypal Analysis and Pareto Task Inference
Author: Philipp Schäfer
Leoni Zimmermann
Paul Burmedi
Avia Walfisch
Noa Goldenberg
Shira Yonassi
Einat Tamar
Miri Adler
Jovan Tanevski
Ricardo Ramirez Flores
Julio Saez-Rodriguez

Dear Dr Ramirez Flores,

Thank you for the submission of your manuscript to Molecular Systems Biology. I have now had a chance to carefully read your manuscript and revision plan. We think the study is of potential interest, and we would like to invite a major revision of your manuscript.

We think your overall revision plan is reasonable. Reviewer #2 noted that the current study remains largely technical and that additional biological applications would significantly strengthen the framework's broader applicability. While we understand the rationale for not pursuing analyses of transient or plastic states, it will be important to incorporate more biological applications in cross-condition comparisons as Reviewer #2 suggested, to enhance the overall impact of the study and make it more suitable for the broader readership of Molecular Systems Biology.

As you may already know, our editorial policy allows in principle a single round of major revision, and it is therefore essential to respond to the reviewers' comments that are as complete as possible. Please feel free to contact me in case you would like to discuss in further detail any of the issues raised by the reviewers.

On a more editorial level, we would ask you to address the following issues:

- Please provide a .docx formatted version of the manuscript text (including legends for main figures, EV figures and tables). Please make sure that the changes are highlighted to be clearly visible.

- Please provide individual production quality figure files as .eps, .tif, .jpg (one file per figure). When assembling figures, please refer to our figure preparation guideline in order to ensure proper formatting and readability in print as well as on screen: https://media.springernature.com/original/springer-cms/rest/v1/content/27825798/data/v1

-Please provide a .docx formatted letter INCLUDING the reviewers' reports and your detailed point-by-point responses to their comments. As part of the EMBO Press transparent editorial process, the point-by-point response is part of the Review Process File (RPF), which will be published alongside your paper.

-Please note that all corresponding authors are required to supply an ORCID ID for their name upon submission of a revised manuscript.

-We replaced Supplementary Information with Expanded View (EV) Figures and Tables that are collapsible/expandable online. A maximum of 5 EV Figures can be typeset. EV Figures should be cited as 'Figure EV1, Figure EV2" etc... in the text and their respective legends should be included in the main text after the legends of regular figures.

Additional Tables/Datasets should be labeled and referred to as Table EV1, Dataset EV1, etc. Legends have to be provided in a separate tab in case of .xls files. Alternatively, the legend can be supplied as a separate text file (README) and zipped together with the Table/Dataset file.

For the figures and tables that you do NOT wish to display as Expanded View figures, they should be bundled together with their legends in a single PDF file called *Appendix*, which should start with a short Table of Content. Each legend should be below the corresponding Figure/Table in the Appendix. Appendix figures and tables should be referred to in the main text as: "Appendix Figure S1, Appendix Figure S2, Appendix Table S1" etc. See detailed instructions regarding expanded view here: https://link.springer.com/journal/44320/submission-guidelines#cms-Expanded-View-data.

-Before submitting your revision, primary datasets (and computer code, where appropriate) produced in this study need to be deposited in an appropriate public database.
Please remember to provide a reviewer password if the datasets are not yet public.
The accession numbers and database should be listed in a formal "Data Availability" section (placed after Materials & Method) that follows the model below (see also https://link.springer.com/partners/embo-press/editorial-policies#Data%20availability%20statement). Please note that the Data Availability Section is restricted to new primary data that are part of this study.
Data availability
The datasets (and computer code) produced in this study are available in the following databases:
- RNA-Seq data: Gene Expression Omnibus GSE46843 (https://www.ncbi.nlm.nih.gov/geo/query/acc.cgi?acc=GSE46843)
- [data type]: [name of the resource] [accession number/identifier/doi] ([URL or identifiers.org/DATABASE:ACCESSION])
*** Note - All links should resolve to a page where the data can be accessed. ***

- At EMBO Press we ask authors to provide source data for the main manuscript figures. You will receive a separate email with instructions for providing source data with your revised manuscript, including how to upload and organize the files.
Additional information on source data and instruction on how to label the files are available < https://link.springer.com/journal/44320/submission-guidelines#cms-Source-data >

- Our journal encourages inclusion of *data citations in the reference list* to directly cite datasets that were re-used and obtained from public databases. Data citations in the article text are distinct from normal bibliographical citations and should directly link to the database records from which the data can be accessed. In the main text, data citations are formatted as follows: "Data ref: Smith et al, 2001". In the Reference list, data citations must be labeled with "[DATASET]". A data reference must provide the database name, accession number/identifiers and a resolvable link to the landing page from which the data can be accessed at the end of the reference. Further instructions are available at < https://link.springer.com/journal/44320/submission-guidelines#cms-Reference-guidelines >.

- We updated our journal's competing interests policy in January 2022 and request authors to consider both actual and perceived competing interests. Please review the policy (https://link.springer.com/partners/embo-press/editorial-policies#Competing%20interest%20disclosures) and update your competing interests if necessary.
Please use the heading "Disclosure statement and competing interests".

- All Materials and Methods need to be described in the main text using our 'Structured Methods' format. According to this format, the Methods section includes a Reagents and Tools Table (listing key reagents, experimental models, software and relevant equipment and including their sources and relevant identifiers) followed by a Methods and Protocols section describing the methods, ideally using a step-by-step protocol format. The aim is to facilitate adoption of the methodologies across labs.

Please download and fill our Reagents and Tools Table template (.docx), which you can find in our author guidelines: https://link.springer.com/journal/44320/submission-guidelines#structuredmethods.

When submitting your revised manuscript, please do not include the Reagents and Tools Table in the Methods section of the manuscript but upload it as a separate file choosing the file type "Reagent Table".

-Regarding data quantification:
Please ensure to specify the name of the statistical test used to generate error bars and P values, the number (n) of independent experiments (please specify technical or biological replicates) underlying each data point and the test used to calculate p-values in each figure legend. Discussion of statistical methodology can be reported in the materials and methods section, but figure legends should contain a basic description of n, P and the test applied.
Graphs must include a description of the bars and the error bars (s.d., s.e.m.).
Please also include scale bars in all microscopy images.

- Please provide a "standfirst text" summarizing the study in one or two sentences (approximately 250 characters, including space), three to four "bullet points" highlighting the main findings and a "synopsis image" (550px width and 400-600 px height, PNG format) to highlight the paper on our homepage.
Please refer to published papers for examples.

When you resubmit your manuscript, please download our CHECKLIST (https://media.springernature.com/original/springer-cms/rest/v1/content/27825796/data/v1) and include the completed form in your submission.
Please note that the Author Checklist will be published alongside the paper as part of the transparent process .

If you feel you can satisfactorily deal with these points and those listed by the referees, you may wish to submit a revised version of your manuscript. Please attach a covering letter giving details of the way in which you have handled each of the points raised by the referees. A revised manuscript will be once again subject to review and you probably understand that we can give you no guarantee at this stage that the eventual outcome will be favorable.

I look forward to receiving the revised manuscript soon.

Kind regards,
Jingyi

Jingyi Hou, PhD
Senior Editor
Molecular Systems Biology

We realize that it is difficult to revise to a specific deadline. In the interest of protecting the conceptual advance provided by the work, we recommend a revision within 3 months (9th Jan 2026). Please discuss the revision progress ahead of this time with the editor if you require more time to complete the revisions.

*** PLEASE NOTE *** As part of the EMBO Press transparent editorial process initiative (see our Editorial at https://dx.doi.org/10.1038/msb.2010.72), Molecular Systems Biology publishes online a Review Process File with each accepted manuscripts. This file will be published in conjunction with your paper and will include the anonymous referee reports, your point-by-point response and all pertinent correspondence relating to the manuscript. If you do NOT want this File to be published, please inform the editorial office at contact@molsystbiol.org within 14 days upon receipt of the present letter.

***
Rev_Com_number: RC-2025-03221
New_manu_number: MSB-2025-13511-T
Corr_author: Ramirez Flores
Title: ParTIpy: A Scalable Framework for Archetypal Analysis and Pareto Task Inference

Dear Dr. Hou,

Thank you for the opportunity to submit our revised manuscript for consideration in *Molecular Systems Biology.* We have revised our software and manuscript to address the reviewers' feedback, with a particular focus on improving the documentation and usability of the ParTIpy package, clarifying the methodological strategy used to characterize the archetypes, providing additional benchmarking analyses supporting our scalability claims, and incorporating additional biological analyses that illustrate the interpretative power of Pareto Task Inference (ParTI).

Specifically we have:

- Fixed the issues downloading example data to run tutorials and clarified processing steps in the documentation.
- Provided an implementation of a quantile-based enrichment analysis originally used to characterize archetypes in the previous implementations of ParTI and compared its results with our proposed method for characterization.
- Benchmarked the memory usage and running times of our proposed framework and our implementation of archetypal analysis to support scalability claims.
- Benchmarked the biological stability of the inference of archetypes using coresets by comparing across datasets to inferences based on complete data.
- Added a new results section focused on the use of ParTIpy in single-cell cross-condition data, showing how transcriptional differences between fibroblasts in failing and non-failing human hearts can be interpreted as shifts in the usage of existing cellular tasks.

We believe that these changes improve the manuscript, in particular convey our message more clearly and our extended analysis demonstrates the robustness of our framework.

We have also fulfilled the formatting and editorial requests (formatted Extended View Figures and tables, and Appendix; formatted Methods section and filled out reagents

table;provided source data for plots when feasible; filled out checklist).

Below is a point-by-point response in green to individual reviewer comments. Sections in the manuscript with major changes are marked in color blue. We hope that our manuscript is now suitable for publication in *Molecular Systems Biology*.

Sincerely,
Julio Saez-Rodriguez and Ricardo O. Ramirez Flores
on behalf of all the authors

Reviewer #1

Reviewer #1 (Evidence, reproducibility and clarity (Required)):

Summary

The paper "ParTIpy: A Scalable Framework for Archetypal Analysis and Pareto Task Inference" presents ParTIpy, an open-source Python package that modernizes and scales the Pareto Task Inference (ParTI) framework for analyzing biological trade-offs and functional specialization. Unlike the earlier MATLAB implementation, which required a commercial license and was limited in scalability, ParTIpy leverages Python's open ecosystem and integration with tools such as scverse to make archetypal analysis more accessible, flexible, and compatible with modern biological data workflows. Through advanced optimization and coreset algorithms, it efficiently handles large scale single cell and spatial transcriptomics datasets. ParTIpy identifies "archetypes", or optimal phenotypic extremes, to reveal how cells balance competing functional programs. The paper demonstrates its application in modeling hepatocyte specialization across the liver lobule, highlighting spatial patterns of metabolic division of labor.
Overall, ParTIpy represents a modern, accessible, and scalable Python-based solution for exploring biological trade-offs and resource allocation in high-dimensional data. The paper is clearly written and addresses an important methodological gap. However, the enrichment analysis differs from the original ParTI framework and should be discussed more explicitly, and the documentation and tutorials, while helpful, could be refined to improve usability and reproducibility.

Major Comments

1. The archetype enrichment analysis used in this paper differs from the original enrichment analysis implemented in ParTI. This is acceptable, but:
a. The authors should explicitly state and discuss the differences between the two approaches.
b. The enrichment analysis should be made more systematic. For each tested feature (e.g. gene or pathway), the analysis should report a p-value for the hypothesis that the feature is enriched near an archetype - that is, its expression (or value) is high close to the archetype and decreases with distance. Appropriate multiple-hypothesis correction should also be applied.

We thank the reviewer for this comment and agree that the differences between our enrichment strategy and the original ParTI MATLAB implementation should be addressed more explicitly.

To address this point, we have implemented the original quantile-based, distance-ranking enrichment procedure within ParTIpy (Method 5.11; pt.compute_quantile_based_gene_enrichment, pt.compute_quantile_based_continuous_enrichment, pt.compute_quantile_based_categorical_enrichment) and now explicitly contrast it with our kernel-based aggregation approach in the revised manuscript, as described below. In

addition, we quantitatively compared both methods and demonstrated their high concordance (Appendix Figure S7).

In Results section **3.3 Characterizing the archetypes**

"[...] Alternatively, ParTIpy implements the quantile-based distance-ranking enrichment algorithm used in previous ParTI work (Hart et al., 2015; Korem et al., 2015; Adler et al., 2019) (see Section 5.11). This approach ranks cells by proximity to each archetype, partitions them into quantile bins, and tests whether gene expression is significantly elevated in the bin closest to the archetype relative to a defined background (by default all other cells; using Mann–Whitney U test). While this procedure produces archetype-specific gene lists, it is computationally demanding because it requires repeated hypothesis testing for each gene–archetype pair. In practice, the kernel-based aggregation of z-scores described above yields highly similar gene rankings (see Appendix Figure S7) while being substantially more time-efficient on large datasets."

In Appendix Figure S7

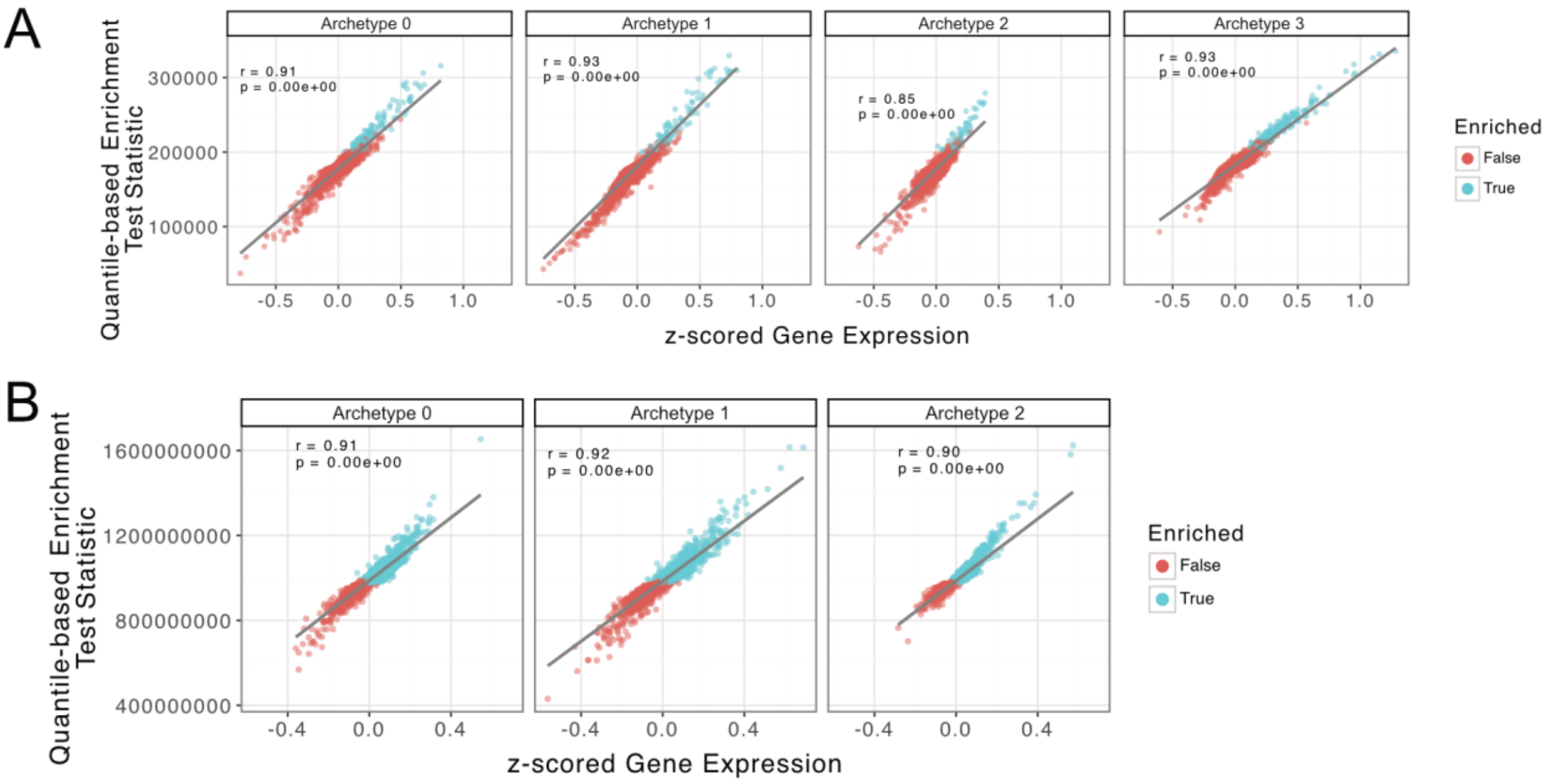

Appendix Figure S7. Concordance between kernel-based archetype expression aggregation and quantile-based enrichment statistics. For each archetype, each point represents a gene. The x-axis shows the archetype-specific aggregated gene-expression z-score obtained by distance-weighted kernel aggregation of single-cell expression profiles (Method Section 5.7). The y-axis shows the corresponding test statistic from the quantile-based distance-ranking enrichment analysis comparing cells closest to the archetype to a background set ((Method Section 5.11); default: Mann–Whitney U test). Points are colored by whether the gene is called enriched by the quantile-based procedure. The gray line indicates the least-squares fit; insets report Pearson's correlation and its two-sided p-value (analytic null distribution, scipy.stats.pearsonr) between the two quantities. (A) Hepatocyte scRNA-seq dataset (Ben-Moshe et al., 2022) analyzed in Section 5.12. (B) Cardiac fibroblast snRNA-seq dataset (Chaffin et al., 2022) analyzed in Section 5.13.

In Methods Section 5.11

“5.11 Quantile-based Enrichment Analysis

As a complementary enrichment strategy aligned with prior ParTI studies (Hart et al., 2015; Korem et al., 2015; Adler et al., 2019), we implement a distance-ranking–based enrichment framework that directly links feature specificity to proximity to archetypes. This approach follows the original ParTI reference implementation.
For a fixed archetype k, we compute the Euclidean distance between the archetype position z_k *and the low-dimensional representation* x_n *of each cell* n*,*

$$d_{kn} = \|\mathbf{z}_k - \mathbf{x}_n\|_2.$$

Cells are ordered by increasing distance to the archetype, and distances are converted to ranks to ensure robustness to absolute distance scale.
Ranked cells are partitioned into disjoint quantile bins of approximately equal size, with each bin containing a fixed fraction of the cells (default: 10%). Larger datasets permit finer stratification while maintaining sufficiently large bin sizes to ensure statistical robustness. Enrichment is evaluated by contrasting a foreground set of cells closest to the archetype with a background set consisting of all remaining cells, although alternative contrasts such as closest versus furthest bins are also supported.

For continuous features such as gene expression, enrichment is assessed by contrasting foreground and background cells using a Mann–Whitney U test by default, with the option to apply Welch’s two-sample t-test. Multiple testing across features and archetypes is controlled using the Benjamini–Hochberg procedure. As an additional specificity criterion, features can be required to attain maximal mean or median expression in the bin closest to the archetype. This requirement is most appropriate for continuous covariates such as age or pseudotime, but may be unstable for sparse features such as gene expression with a high fraction of zero values.

For categorical features (e.g. disease status or sex), each category level is treated as a one-versus-rest indicator variable. Enrichment is evaluated by comparing category frequencies across quantile bins, defining an enrichment curve as the ratio between the category frequency within each bin and its global frequency across all cells. Categories are required to show maximal enrichment in the bin closest to the archetype. Statistical significance of over-representation in the foreground relative to the background is assessed using a hypergeometric test, with multiple testing correction performed using the Benjamini–Hochberg procedure.”

In addition, we clarify that pathway-level enrichment analyses performed using decoupler-py report two-sided p-values derived from the univariate linear model (ULM), with multiple testing controlled using the Benjamini–Hochberg procedure, as illustrated in Figure 3E and Figure 4G-H, where BH-adjusted ULM p-values are reported. Given the strong concordance between gene rankings obtained from the quantile-based enrichment framework and from the kernel-based aggregation of z-scored gene expression (Appendix Figure S7), pathway-level enrichment based on ULM is expected to yield results consistent with the quantile-based strategy described in previous ParTI work (Hart et al., 2015), which effectively applies the same distance-ranking approach to the mean expression of genes within one gene set.

Importantly, the ULM framework further enables the use of weighted and directed gene sets, such as transcription factor regulons or pathway footprint models, in which genes contribute with positive or negative weights, thereby extending the enrichment analysis beyond unweighted gene-set averaging which has proven to be useful in other settings (Badia-I-Mompel, et. al., 2022).

In Methods section 5.8

"***5.8 Functional Enrichment Analysis***

To interpret archetype-specific transcriptional programs, we performed functional enrichment analysis on the archetype-aggregated expression profiles using decoupler-py (Badia-i-Mompel et al., 2022). By default, we applied the univariate linear model (ULM), which relates archetype expression profiles to predefined gene sets or regulons according to

$$\mathbf{y}_k^{(Z)} = \beta_0 + \beta_1 \mathbf{w} + \boldsymbol{\varepsilon}.$$

Here, $y_k{}^{(Z)}$ denotes the expression profile of archetype k and w encodes gene-set or regulon weights. Intuitively, if genes with larger weights tend to be more highly expressed in archetype k, the estimated coefficient β_1 will be positive and large in magnitude. For unweighted gene sets, such as MSigDB's Hallmark gene sets (Subramanian et al., 2005; Liberzon et al., 2015), weights are binary and indicate gene-set membership. Enrichment scores are derived from the estimated effect β_1, and statistical significance is assessed using the associated t-statistic. P-values are corrected for multiple testing using the Benjamini–Hochberg procedure."

2. The package documentation on GitHub and ReadTheDocs is a major strength, but the tutorials can be improved for clarity and accessibility:

We thank the reviewer for this positive feedback. Indeed, providing comprehensive documentation to facilitate ease of adoption was a major motivation behind this project. In response to the reviewer's suggestions, we have revised the tutorials to further improve their clarity, structure, and accessibility, as detailed below.

a. The documentation should list external dependencies that need to be installed seperately, e.g. pybiomart.

We thank the reviewer for pointing this out. We had added all dependencies under the optional-dependencies.extra header, which allows users to run `pip install partipy[extra]` to be able to run all tutorial notebooks. However, we forgot to explain that in the tutorial or Readme page, which we corrected now. The Readme now reads:

```
Install the latest stable full release from PyPI with the extra
dependencies (e.g., `pybiomart`, `squidpy`, `liana`) that are
required to run every tutorial:

```
```
```

```
pip install partipy[extra]
```

Additionally we include clarifications in every tutorial notebook that uses additional dependencies: “To run this notebook, install ParTIpy with the tutorial extras: `pip install partipy[extra]`”.

b. The dataset used in the Quickstart demo appears to be inaccessible or extremely slow to download (the function load_hepatocyte_data_2() did not complete even after 30 minutes, at least in my experience). The authors should verify data availability on Zenodo and consider providing a smaller or cached version to make the demo more reliable and reproducible.

We thank the reviewer for this helpful comment. We agree that the previous implementation of load_hepatocyte_data_2() was not reliable due to slow download speeds from Zenodo. To address this, we now host the required AnnData object on figshare (https://figshare.com/articles/dataset/scRNA-seq_hepatocyte_data_from_Ben-Moshe_et_al_2022_/30588713?file=59459459), ensuring faster and more stable access for the Quickstart tutorial via `scanpy.read`:

```
adata = sc.read("data/hepatocyte_processed.h5ad",
backup_url="https://figshare.com/ndownloader/files/59459459")
```

c. The tutorial order could be more intuitive - for instance, "archetype crosstalk network" appears before "archetypal analysis". Consider starting with the simulated dataset and presenting the full pipeline before moving to more complex real-world examples.

We thank the reviewer for this helpful suggestion and agree that the previous ordering was not intuitive. We have reordered the tutorials such that the notebook introducing archetypal analysis now appears first, followed by the Quickstart tutorial and the subsequent applied examples.

Minor comments

1. In the Python function, the parameter "optim" could use more descriptive option names - for example, renaming "projected_gradients" to "PCHA" would make it clearer and more consistent with terminology used in the paper.

We thank the reviewer for this helpful suggestion. We agree that the previous naming could be misleading. While PCHA does not precisely describe the underlying algorithm, it is the term most users are familiar with from the literature. We have therefore updated the function to accept both "PCHA" and "projected_gradients", which now map to the same underlying optimization routine.

2. In the Quickstart preprocessing, the authors use the following code:
sc.pp.normalize_total(adata)
sc.pp.log1p(adata)
However, they do not specify the target sum in the normalize_total function. The authors should ensure that the data values before the logarithmic transformation span several orders

of magnitude (e.g., 0-10,000); if normalization is performed to a sum of 1, the log transformation becomes ineffective.

By default, sc.pp.normalize_total scales the counts in each cell to the median total counts across all cells, which preserves the typical range of expression values prior to logarithmic transformation. We therefore consider this default behavior appropriate for the Quickstart example. To clarify this point, we have now explicitly stated in the Quickstart tutorial: “By default, normalize_total scales each cell to the median total counts across all cells, which preserves the typical range of expression values prior to logarithmic transformation.”

Referee cross-commenting

I agree with Reviewer #2 observation that the paper's contribution is primarily technical; however, I consider this technical advance to be an important and timely one that will enable many biologists to apply archetypal analysis more effectively in their own work.

We thank the reviewer for this positive and encouraging assessment.

Reviewer #1 (Significance (Required)):

This study presents ParTIpy, a Python-based implementation of Pareto Task Inference (ParTI) that makes archetypal analysis more accessible, scalable, and compatible with modern single-cell and spatial transcriptomics workflows. Its main strength lies in translating a conceptually powerful but technically limited MATLAB framework into an open-source, efficient Python package, enabling wider use in computational biology. The package is well-documented, which further enhances its accessibility and adoption potential, though documentation could be improved to enhance reproducibility and ease of use. It will be of interest to computational systems biologists, particularly those working with omics data, and those interested in studying functional trade-offs and resource allocation.

We appreciate the reviewer’s positive evaluation and are encouraged by their recognition of ParTIpy’s relevance and potential impact in computational biology.

Reviewer #2

Reviewer #2 (Evidence, reproducibility and clarity (Required)):

Summary

This paper introduces the software ParTIpy, a scalable Python implementation of Pareto Task Inference (ParTI), designed to infer functional trade-offs in biological systems through archetypal analysis. The framework modernizes the previous toolbox with efficient optimization, memory-saving coreset construction, and integration with the scverse ecosystem for single-cell transcriptomic data.

Using hepatocytes scRNA-seq data as a test case, the authors identify archetypes corresponding to distinct gene expression patterns. These archetypes align with known liver domains in spatial transcriptomics data, validating both the method's interpretability and its biological relevance.

Major comments

(1) Conclusions
The core computational and biological claims are well supported. ParTIpy clearly scales better than earlier implementations and reproduces known biological structure. However, claims about "scalability to large datasets" should be further qualified (see below).

We thank the reviewer for this constructive comment and agree that the original version did not sufficiently support the scalability claims with large-scale benchmarks. In response, we have added an additional benchmark including datasets with up to two million cells and expanded the evaluation of coresets. These additions are described in detail below.

(2) Claims
Archetypal analysis based on current matrix computation formulation is non-parametric, and new data require recomputation of archetypes. Therefore, the method cannot generalize to unseen data in the way deep learning approaches, which could be further acknowledged and clarified.

We agree that our original Discussion did not adequately address the distinction between classical archetypal analysis, which requires refitting when new data are added, and deep archetypal analysis, which learns an amortized mapping from observations to archetypal coordinates. We have revised the Discussion accordingly by adding the following paragraph.

"[...] One notable extension of archetypal analysis is deep archetypal analysis (van Dijk et al., 2019; Keller et al., 2020; Wang and Zhao, 2022; Milite, Caravagna and Sottoriva, 2025), which replaces the direct optimization of coefficient matrices with an encoder-decoder network. The encoder learns a posterior distribution for the latent representation of the data (X) and for the coefficient matrices A, B which define the archetypes ($Z = BX$) and reconstruction ($\hat{X} = AZ$). This approach captures non-linear structures in the data and achieves scalability to large datasets through mini-batch optimization and amortized

variational inference. While amortized inference provides a direct mapping from observations to archetypal coordinates, enabling fast inference once the model is trained, it can introduce an amortization gap, so that the predicted archetypal coordinates are suboptimal compared to those obtained by directly optimizing variational parameters (Cremer, Li and Duvenaud, 2018; Margossian and Blei, 2024). In contrast, ParTIpy achieves scalability through coreset-based optimization, enabling direct parameter inference even for large datasets."

(3) Additional suggested analyses or experiments

1) Absolute performance benchmarks : it's suggested to report wall-clock time and memory for a few dataset sizes (10k, 100k, 1M cells).

We have added a new benchmark to address this point, as described in the paragraph below and in Methods Section 5.4

In Results section

"We additionally quantified absolute wall-clock runtime and peak memory usage across dataset sizes from 10,000 to 2,000,000 cells on three large single-cell datasets (Appendix Figure S6; Table EV3). For one million cells, fitting six archetypes on a 10% coreset required 45–105 seconds (mean across datasets), and coreset optimization consistently reduced runtime across archetype numbers (Appendix Figure S6A–B). Peak memory scales mainly with the in-memory AnnData object, whereas the archetypal-analysis optimization step itself operates on a low-dimensional PCA representation and therefore requires comparatively little memory (Appendix Figure S6C–D)."

Methods Section

"***5.4 Benchmarking Memory and Time Requirements***

Memory and runtime benchmarks were performed on three large single-cell datasets (K562, HCT116, and HEK293T) (Appendix Figure S6; Table EV3). To reduce variability due to parallelization, BLAS thread counts were fixed to one for all benchmark runs.

Peak memory usage was measured using resident set size (RSS), tracked via /proc/self/statm and ru_maxrss, with sampling every 50 ms. To assess scaling behavior, datasets were subsampled to predefined numbers of cells using a fixed random seed (42) to generate reproducible index subsets. Subsampling ranges were 1,000 to 1,000,000 cells for K562 and 1,000 to 2,000,000 cells for HCT116 and HEK293T. Each subsample was processed independently.

Preprocessing consisted of total-count normalization, log1p transformation, selection of highly variable genes (n_top_genes = 2,000), and principal component analysis (PCA). The first 20 principal components were used as the input embedding for archetypal analysis. Benchmarks were conducted across combinations of the number of archetypes (K {3, 6, 9}), coreset fractions ({0.1, 1.0}), and five random seeds. Each benchmark run was executed in a

separate process. For runs with coreset_fraction < 1.0, the standard coreset algorithm was used.

To isolate the memory footprint of the archetypal analysis optimization step itself, we performed an additional PCA-only benchmark in which archetypal analysis was applied directly to precomputed PCA matrices rather than full AnnData objects. Specifically, we extracted the PCA embedding (pca_bench=adata.obsm["X_pca"][:, :20]) and fit an archetypal analysis model directly on this matrix using pt.AA(…).fit(X=pca_bench), instead of invoking pt.compute_archetypes on an AnnData object. This benchmark excludes memory overhead associated with having the full single-cell AnnData object in memory, thereby attributing observed memory usage specifically to the optimization procedure operating on the low-dimensional embedding.
"

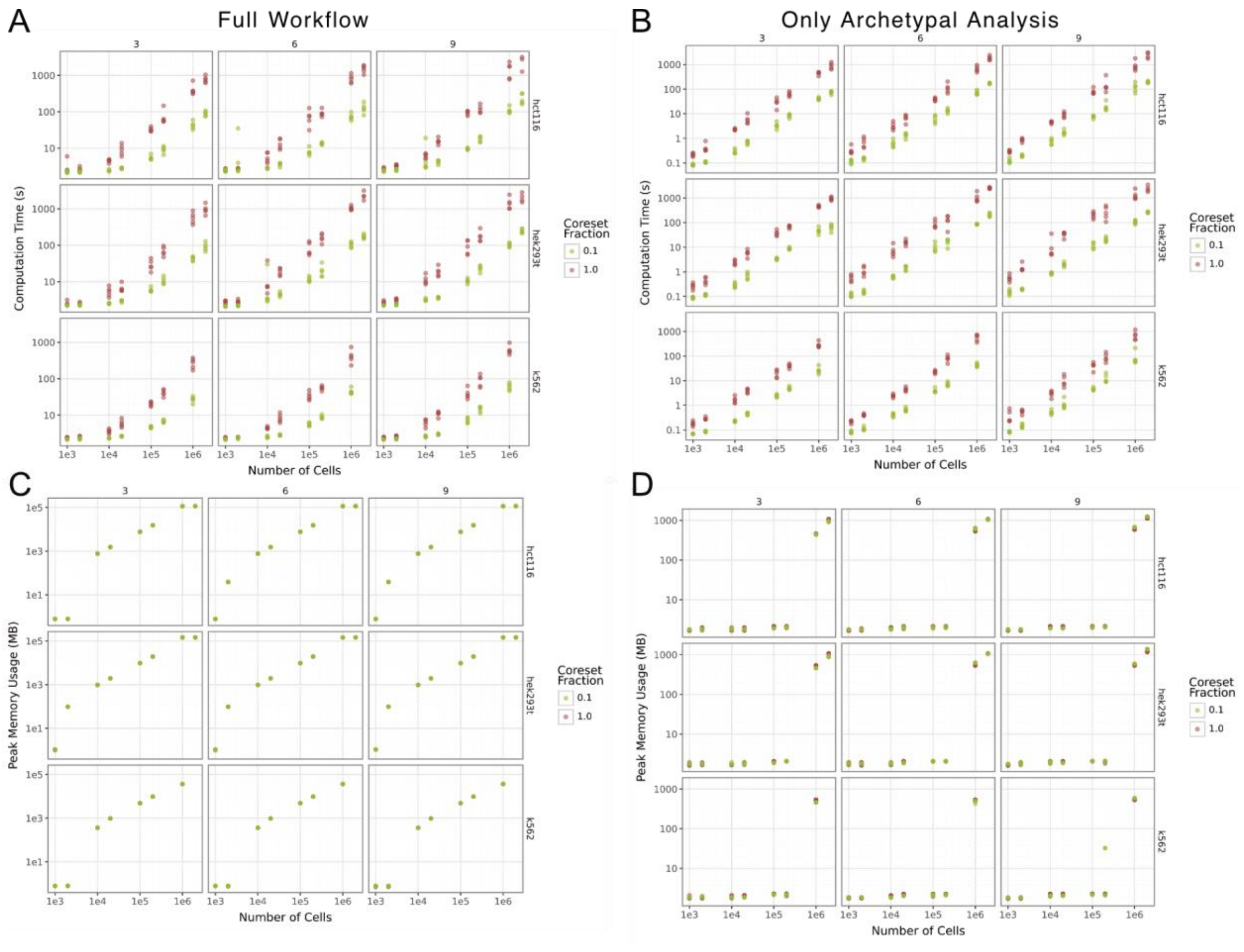

Appendix Figure S6. *Wall-clock time and peak memory scaling of ParTIpy. (A) Wall-clock runtime (seconds) of the full ParTIpy workflow as a function of the number of cells, evaluated on three large single-cell datasets (rows: HCT116, HEK293T, K562; see Supplementary Table S3). Columns indicate the number of archetypes (3, 6, 9). Each point corresponds to an independent run at a given dataset size (log–log axes). Colors denote optimization on the full dataset (coreset fraction = 1.0) or on a 10% coreset (coreset fraction = 0.10). Runtime increases monotonically with dataset size and is consistently reduced by coreset optimization across datasets and archetype numbers. (B) Wall-clock runtime for the archetypal analysis optimization step alone, isolating the computational cost of fitting archetypes from preprocessing and data handling. (C) Peak memory usage (MB) for the full workflow (log–log axes). Memory consumption scales primarily with the number of cells due to retaining the full AnnData object in memory. (D) Peak memory usage for archetypal analysis optimization alone. The optimization step has a small memory footprint, as it operates exclusively on a low-dimensional PCA representation of the data rather than on the full feature matrix.*

2) Coreset sensitivity analysis: Could authors show how coreset size affects archetype positions and biological interpretation?

We have extended the coreset benchmark to evaluate the effect of coreset size on archetype stability and biological interpretation, as described below.

In Results Section

“Furthermore, we verified that coreset-based archetypes captured the same biological signal as full-data optimization. Specifically, we aligned coreset and full-data archetypes using Hungarian matching on pairwise Euclidean distances between archetype locations, and then compared the resulting archetype-specific gene-expression profiles. Across coreset fractions of >1% of cells, these profiles remained highly concordant, with a median Pearson’s correlation above 0.9 (Appendix Figure S5).”

In Methods Section

“5.3 Benchmarking Coresets

Coreset benchmarking was performed on the same datasets used for the initialization and optimization benchmarks (see Section 5.2). We evaluated coreset fractions of 0.64, 0.32, 0.16, 0.08, 0.04, 0.02, 0.01, and 0.005.

For each dataset, reference archetypes were computed on the full data using five random restarts (n_restarts = 5) with a fixed random seed (42). Using these reference archetypes, we computed archetype weights and z-scaled archetype expression profiles. For each coreset fraction and random seed (MS snRNA- seq: 20 seeds; MS Xenium: 20 seeds; lupus PBMC: 20 seeds), archetypal analysis was then performed using the default coreset algorithm (coreset_algorithm = “standard”).

Archetypes inferred from each coreset run were aligned to the corresponding reference archetypes using Hungarian matching based on pairwise Euclidean distances between archetype locations. To quantify agreement between coreset- and full-data solutions, we computed the Pearson correlation between archetype-specific characteristic expression profiles, as well as the variance explained by the archetypal reconstruction. [...]”

Referee cross-commenting

I agree with the other reviewer's suggestion to check consistency and reproducibility with previous implementation, and enhance the tutorial of the software for users from a biological background. Combined with my comments to further improve the biological application showcase, the revised manuscript could be an impactful contribution to the field, if these comments could be properly addressed.

Reviewer #2 (Significance (Required)):

(1) Advance

This paper is primarily a technical contribution. It modernizes the Pareto Task Inference framework into a scalable and user-friendly Python implementation, which is valuable. However, to further improve its significance especially for the broader biological audience, more detailed analysis could be performed (see below)

(2) Biological scope and applications [optional]

The current biological validation in hepatocyte is technically fine but limited in breadth and impact. It demonstrates that ParTIpy works but falls in short of showing what new insights it can reveal. Several promising applications could be further explored:

1) Cross-condition comparisons: could ParTIpy quantify how the Pareto front shifts between conditions (e.g., normal vs. tumor, treated vs. control)?

We thank the reviewer for this valuable suggestion. In response, we have added a new Results section demonstrating how ParTIpy can quantify cross-condition shifts in cellular task allocation. Specifically, we analyze 147,000 fibroblasts from human adult heart tissue comprising 16 non-failing (NF) donors and 26 cardiomyopathy (CM) donors. We find that cardiomyopathy induces a significant redistribution of fibroblasts within a shared three-archetype task space, with cardiomyopathy donors shifted toward an activated fibrotic program (archetype 2) and non-failing donors closer to a stress-responsive/quiescent program (archetype 0). Importantly, these shifts reflect altered allocation among pre-existing archetypal programs rather than the emergence of disease-specific cell states.

Additionally, this case study is now provided as a fully reproducible tutorial in the ParTIpy documentation:
https://partipy.readthedocs.io/en/latest/notebooks/cross_condition_fibroblast.html

In Results Section

"3.5 Using ParTIpy to Quantify Cross-Condition Shifts in Cellular Task Allocation

Cross-condition analyses are a central component of single-cell transcriptomic studies and are commonly performed by discretizing cells into clusters followed by tests for condition-dependent changes in cluster abundance or gene expression. While effective in many settings, such approaches impose sharp boundaries on cell states that are often intrinsically continuous. ParTIpy enables an alternative formulation by modeling cells as continuous mixtures of archetypal programs ("tasks"), thereby enabling the quantification of condition-dependent transcriptional shifts within a shared task space.

To illustrate this workflow, we analyzed single-nucleus RNA-seq data from human adult heart tissue comprising 16 non-failing (NF) donor hearts and 26 cardiomyopathy (CM; pooled hypertrophic and dilated cardiomyopathy) hearts, focusing on approximately 147,000 fibroblasts (Figure 4A) (Chaffin et al., 2022). Fibroblasts are particularly well suited for this analysis, as they exhibit pronounced disease-associated transcriptional remodeling and

span a continuum from quiescent ECM maintenance to activated, fibrotic programs in cardiomyopathy (Patrick et al., 2024; Lanzer et al., 2025; Youness et al., 2025).

Following normalization, dimensionality reduction, and donor-level batch correction using Harmony (Korsunsky et al., 2019) (see Methods), we determined the dimensionality of the archetypal analysis input using a shuffled PCA baseline (see Methods) and retained the first 16 principal components (Figure EV2A). We then evaluated the number of archetypes using the complementary criteria implemented in ParTIpy (Figure EV2B–D). While both variance explained and the information criterion favored a four-archetype solution, this configuration yielded one archetype with high positional variance across bootstrap replicates, indicating limited robustness. We therefore selected a three-archetype model, which showed reduced reconstruction but substantially improved bootstrap stability and reproducibility, enabling a more robust and interpretable biological characterization (Figure 4B, Figure EV2D–E).

To quantify the shift in task allocation during cardiomyopathy, we aggregated cells into donor-level pseudobulks by averaging the Harmony-corrected principal component coordinates of all fibroblasts within each donor (Figure 4C). This avoids pseudo-replication and provides a concise summary of the mean fibroblast task allocation per patient. To formally assess these differences, we computed Euclidean distances from each donor-level pseudobulk to each archetype and compared distances between cardiomyopathy and non-failing donors using two-sided Welch's t-tests. Distances to all three archetypes differed significantly between conditions (Figure 4D). These differences were most pronounced for archetype 0, which was closer to non-failing donors, and archetype 2, which was closer to cardiomyopathy donors, whereas shifts along archetype 1 were comparatively weaker.

To interpret the biological meaning of the inferred archetypes, we characterized their transcriptional profiles using the aggregated z-scored gene expression together with downstream estimation of transcription factor activities and gene-set enrichment analyses. Archetype 2, which was preferentially enriched in cardiomyopathy donors, corresponded to an activated fibrotic program, marked by relatively higher expression of canonical fibrotic ECM genes including POSTN, FN1, COL1A1, VCAN, TNC, and LOXL1 (Figure 4E) (Fu et al., 2018; Torimoto et al., 2024). Consistently, this archetype showed strong activation of mesenchymal and mechanotransduction-associated transcription factors such as TWIST1 (Wei et al., 2015), SCX (Bagchi et al., 2016), PRRX1 (Lee et al., 2022), and SRF (Olson and Nordheim, 2010), as inferred from CollecTRI (Müller-Dott et al., 2023) regulons using decoupler ULM (Badia-i-Mompel et al., 2022) (Figure 4F). At the pathway level, archetype 2 was enriched for MSigDB NABA Matrisome gene sets (Naba et al., 2016) related to the core matrisome, collagens, ECM glycoproteins, and proteoglycans, reflecting broad extracellular matrix remodeling (Figure 4G).

In contrast, archetype 1 was characterized by a perivascular and basement-membrane–associated program, with elevated expression of structural ECM components such as COL15A1, COL4A1, and laminins (including LAMB1 and LAMC1) (Gatseva et al., 2019), and comparatively limited activation of fibrotic genes (Figure 4E). This archetype showed selective enrichment of NABA gene sets associated with basement membranes and ECM-affiliated proteins, consistent with a more structural and niche-supporting fibroblast role (Figure 4G).

Archetype 0, which was preferentially enriched in non-failing donors, exhibited features of a quiescent, yet stress-responsive program (Chen et al., 2012). This archetype was characterized by genes involved in hypoxic, inflammatory, and oxidative stress responses and was associated with increased activity of stress-associated transcription factors, including NFKB, HIF1A, NR3C1, CEBPB, and NFE2L2 (Figure 4F). Correspondingly, matrisome enrichment for archetype 0 was limited and depleted from core fibrotic components (Figure 4G).

Although archetype 2 was preferentially enriched in cardiomyopathy, it was not specific to that condition. In every non-failing donor, a relatively small subset of fibroblasts was closer to archetype 2 than to any other archetype (median 115 cells per donor; Figure 4H). Together, these results indicate that cardiomyopathy redistributes cardiac fibroblasts from stress-responsive toward activated fibrotic programs within a shared archetypal space, reflecting altered allocation among existing cellular programs rather than the emergence of disease-specific fibroblast states. More broadly, this example illustrates how ParTIpy enables a principled and interpretable framework for cross-condition analysis in single-cell data by linking low-dimensional geometric structure of gene expression in single cells to biologically meaningful shifts in cellular task allocation."

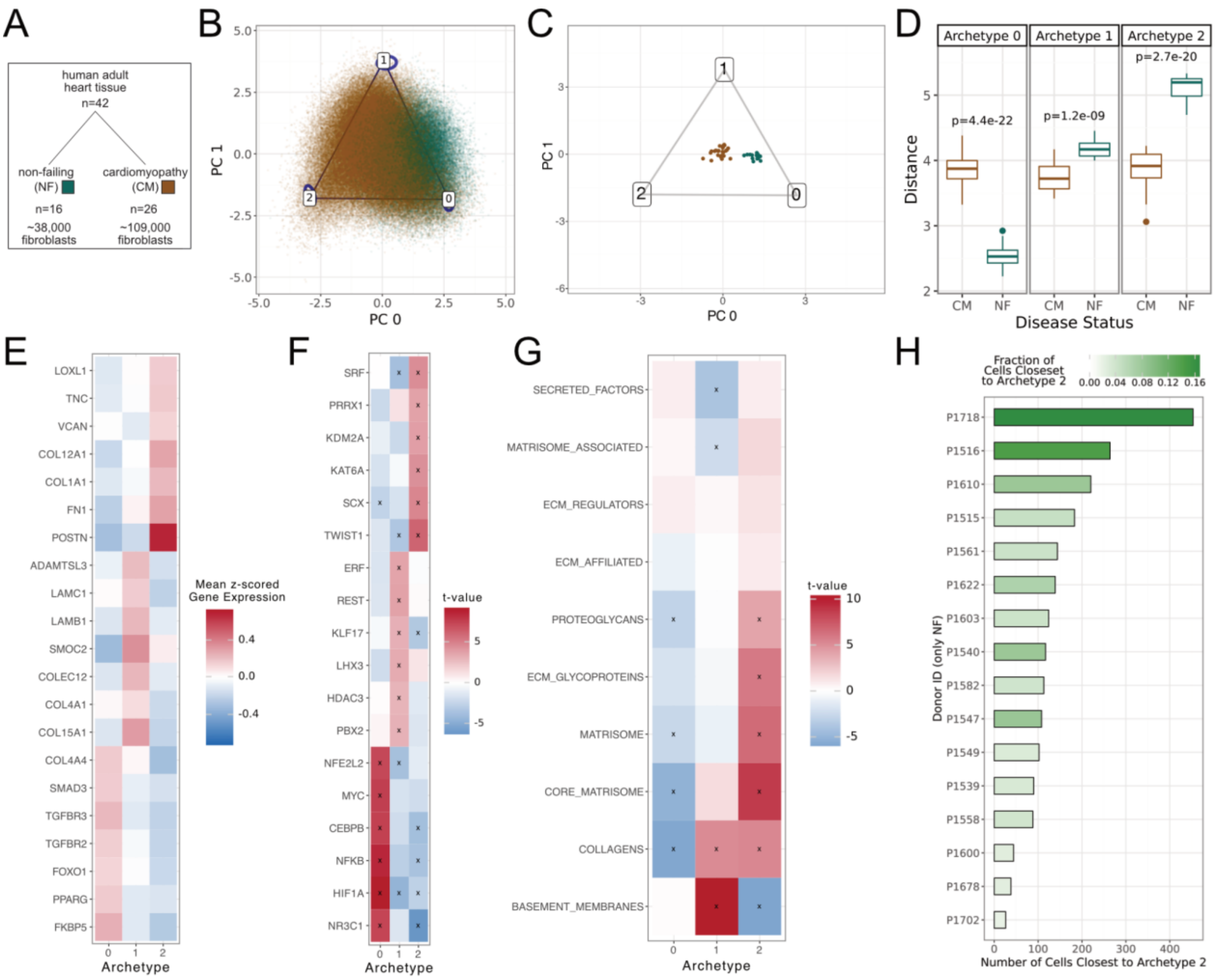

Figure 4. Cross-condition archetypal analysis of human cardiac fibroblasts in non-failing hearts and cardiomyopathy. (A) Study design and sample composition. (B) Archetypal analysis (AA) fit shown in

the first two harmony-integrated principal components. Each point denotes a fibroblast nucleus, colored by disease status. The triangle indicates the inferred K=3 archetypes, representing extreme transcriptional programs that span the observed continuous fibroblast state space. (C) Donor-level pseudobulk profiles in the same PC space and shown within the archetype convex hull, illustrating condition-dependent shifts at the patient level. (D) Donor-level distances to each archetype stratified by disease status. Boxplots summarize donor distributions; distances quantify proximity of each donor's pseudobulk profile to each archetypal program (smaller distance indicates closer proximity). P-values shown are from two-sided Welch's t-tests comparing CM vs non-failing donor distances for each archetype. (E) Expression of curated fibroblast marker genes across archetypes, shown as mean z-scored expression in archetype-aggregated profiles, highlighting archetype-specific ECM remodeling and perivascular/basement-membrane programs. (F) Transcription factor (TF) activity differences across archetypes inferred using the univariate linear model (ULM) implemented in decoupler (Badia-i-Mompel et al., 2022) with the CollecTRI regulon resource (Müller-Dott et al., 2023). ULM estimates TF activity and derives two-sided p-values from a linear model relating archetype-aggregated expression to TF–target weights. P-values are adjusted for multiple testing using the Benjamini–Hochberg procedure; significance marks indicate p_adj ≤ 0.05. (G) Matrisome program activity across archetypes inferred using ULM with MSigDB NABA Matrisome gene sets (Naba et al., 2016). P-values are derived using the ULM framework described in (F). (H) Number of cells per non-failing donor that are closest to archetype 2.

In Methods Section

"5.13 Fibroblast Cross-Condition Analysis

Single-nucleus RNA-seq data from human dilated and hypertrophic cardiomyopathy, together with non-failing donor hearts, were obtained from (Chaffin et al., 2022) (see Table EV2). Cells were subset to fibroblast annotations (Fibroblast_I, Activated_fibroblast, Fibroblast_II). Low-quality cells and lowly expressed genes were filtered using sc.pp.filter_cells (min_genes = 100) and sc.pp.filter_genes (min_cells = 10). Disease labels were collapsed to non-failing (NF) and cardiomyopathy (CM).

Counts were total-count normalized (sc.pp.normalize_total) and log-transformed (sc.pp.log1p). Highly variable genes were selected (sc.pp.highly_variable_genes, default arguments), and principal component analysis (PCA) was computed on the selected HVGs (sc.pp.pca with mask_var = "highly_variable", n_comps = 50). A z-scored expression layer was stored for downstream archetype characterization (sc.pp.scale with max_value = 10). To correct donor-specific effects, batch correction across donors was performed using Harmony (Korsunsky et al., 2019) (hm.run_harmony) on the PCA embedding, yielding a Harmony-integrated space (X_pca_harmony). Archetypal analysis was performed in this Harmony-corrected embedding using the first 16 components, with the effective dimensionality determined using a shuffled PCA baseline (pt.compute_shuffled_pca), and a

K = 3 archetype model was fitted. For the shuffled PCA baseline, PCA was applied to the mean-centered gene-expression matrix (log1p-normalized highly variable genes). To estimate a null distribution, we generated 50 shuffled datasets by independently permuting each gene (column) across cells, thereby preserving marginal gene distributions while removing gene–gene covariance. PCA was fitted to each shuffled matrix, and components were retained if their explained variance in the original data exceeded the shuffled mean plus one standard deviation, resulting in 16 components used for downstream archetypal analysis.

Model selection followed the ParTIpy workflow. The number of archetypes was evaluated using variance explained and information-criterion curves (pt.compute_selection_metrics) together with bootstrap-based archetype stability analysis (pt.compute_bootstrap_variance). Archetype weights were computed using pt.compute_archetype_weights.
For cross-condition comparisons, donor-level pseudobulks were computed by averaging Harmony coordinates across fibroblasts within each donor. Donor pseudobulks were projected into archetype coefficient space (pt.AA(...).transform), and Euclidean distances to each archetype were computed. Disease effects on donor-level distances were assessed using two-sided Welch's t-tests for each archetype.

Archetype expression profiles were computed for raw counts, log1p-normalized values, and z-scored values (pt.compute_archetype_expression), restricting to genes expressed in more than 100 cells. For donor-level summaries of proximity to archetype 2, cells were assigned to their maximum-weight archetype using the coefficient matrix A, and the number of NF cells closest to archetype 2 was tabulated per donor.

Transcription factor (TF) activity was inferred from archetype z-scored expression profiles using decoupler's univariate linear model (ULM) (Badia-i-Mompel et al., 2022). CollecTRI regulons (Müller-Dott et al., 2023) were retrieved (dc.op.collectri with organism = "human") and scored (dc.mt.ulm). Pathway activity was inferred analogously using PROGENy (dc.op.progeny with organism = "human", followed by dc.mt.ulm). NABA Matrisome (Naba et al., 2016) gene sets were extracted from the MSigDB resource (dc.op.resource with resource = "MSigDB"), excluding cancer-specific subsets, and scored using the same ULM workflow. For all ULM-based analyses, two-sided p-values were adjusted using the Benjamini–Hochberg procedure."

In addition, we note that ParTIpy's applicability to cross-condition analyses is further illustrated in our online tutorials (cross-condition lupus example: https://partipy.readthedocs.io/en/latest/notebooks/cross_condition_lupus.html).

2) Transient or plastic states: Cells with mixed archetype weights or high mixture entropy can be interpreted as transient, functionally flexible states. ParTIpy can quantify such transience geometrically, even in static data, which providing a competitive counterpart to models like CellRank or CellSimplex (https://doi.org/10.1093/bioinformatics/btaf119).

We thank the reviewer for this insightful suggestion and agree that this represents an important direction. While interpreting mixed archetype weights as transient or plastic states is conceptually appealing, validating whether such "generalist" cells truly correspond to

transitional states would require complementary temporal information. We have added the following statement to the Discussion:

“When snapshot data capture ongoing cellular transitions (e.g. tissue remodeling), or when densely sampled time-course data are available, integrating estimated cell–cell transition probabilities (Weiler et al., 2024)—derived from RNA velocity, pseudotime algorithms, or optimal transport—could further strengthen interpretation by distinguishing stable task specializations from transient states and by quantifying trajectories within the archetypal simplex (Liu et al., 2025).”

Because the manuscript does not explore these scenarios, the biological impact remains narrow, and the framework's broader interpretive power is somehow underrepresented.

We agree that the original manuscript did not fully illustrate the broader interpretive power of the framework. We believe that the newly added cross-condition application substantially strengthens this aspect by demonstrating how ParTIpy enables principled quantification of condition-dependent shifts in cellular task allocation.

(3) Audience and impact

The paper will interest computational biologists, systems biologists, and bioinformaticians focused on single-cell analysis, and its impact will grow substantially if the authors demonstrate more biological applications.

(4) Reviewer expertise
Computational biology, single-cell transcriptomics, machine learning, computational math

25th Mar 2026

Manuscript Number: MSB-2025-13511R
Title: ParTIpy: A Scalable Framework for Archetypal Analysis and Pareto Task Inference
Author: Philipp Schäfer
Leoni Zimmermann
Paul Burmedi
Avia Walfisch
Noa Goldenberg
Shira Yonassi
Einat Tamar
Miri Adler
Jovan Tanevski
Ricardo Ramirez Flores
Julio Saez-Rodriguez

Dear Ricardo,

Thank you for sending us your revised manuscript. We have now received the enclosed reports from the two reviewers who evaluated the previous version of the manuscript at Review Commons. As you will see below, the reviewers are both satisfied with the revisions made. Before we can formally accept your manuscript, we would ask you to address the following editorial-level issues:

1. Please remove all figures from the manuscript file. The legends for both the main figures and the EV figures should remain at the end of the manuscript, after the References section.

2. Remove "Authors' contribution" section from the manuscript file.

3. Please add the missing callout for Figure 3C.

4. Please note that the source file names for EV figures should follow the format "Figure EV1", "Figure EV2", and so on.

5. Please ensure that the grant information listed in the manuscript matches the information entered in the submission system. In particular, please review grants INST 35/1803-1 FUGG, INST 35/1804-1 LAGG, and INST 35/1597-1 FUGG , as they are currently not included in the system.

6. References: "et al." should be used after 10 author names. DOIs should only be included for preprints and datasets that have not yet been published.

7. Appendix: Please remove the separate file titled "Supplementary Methods" and incorporate all supplementary methods into the Appendix. Additionally, remove the highlights.

8. There are two footnotes on page 3. Could you insert the second one into the manuscript text and remove the footnote? As for the first, is there a specific reason to keep it, or can it be removed as well?

9. Source Data
- Main figures: Provide source data as one ZIP folder per figure. Each folder should contain clearly labeled panels.
- SD for EV figures can be combined into a single set, with nomenclature adjusted to "Figure EV1-EV2."

10. Tables EV1-EV3 should be removed from the manuscript file. EV tables should be uploaded as separate files, with one file per table. Each file should include a separate sheet labeled "Legend" containing the corresponding table legend.

11. Please address the following issues in figure legends:
- Please note that the box plots need to be defined in terms of minima, maxima, centre, bounds of box and whiskers, and percentile in the legend of figure 4D
- Please note that information related to n is missing in the legend of figure 4D
- Figure EV1: Label F is missing from the figure caption, while label N is present. Please review this.

12. The manuscript sections should be in the following order: Title page - Abstract - Introduction - Results - Discussion - Methods - Data Availability - Acknowledgments - Disclosure and Competing Interests Statement - References - Figure Legends -

(Main Tables with legends if applicable) - Expanded View Figure Legends.

As a matter of course, please make sure that you have correctly followed the instructions for authors as given on the submission website.

Thank you for submitting this paper to Molecular Systems Biology.

Kind regards,
Jingyi

Jingyi Hou, PhD
Senior Editor
Molecular Systems Biology

*** PLEASE NOTE *** As part of the EMBO Publications transparent editorial process initiative (see our Editorial at https://www.nature.com/msb/journal/v6/n1/full/msb201072.html), Molecular Systems Biology will publish online a Review Process File to accompany accepted manuscripts. When preparing your letter of response, please be aware that in the event of acceptance, your cover letter/point-by-point document will be included as part of this File, which will be available to the scientific community. More information about this initiative is available in our Instructions to Authors. If you have any questions about this initiative, please contact the editorial office (msb@embo.org).

Reviewer #1:

The authors have addressed all of my previous comments.
In particular, they have clarified and improved the enrichment analysis, strengthened and reorganized the documentation and tutorials, and resolved the issues related to data accessibility and usability. The revisions have significantly improved the clarity, reproducibility, and overall usability of the framework.

Overall, the manuscript is now clear, well-structured, and technically sound. I have no further major concerns and believe it is suitable for publication.

Reviewer #2:

The authors have done impressive work to address my previous questions, especially enhancing the presentation of scalability and the broader biological significance of the package. I have no further questions and endorse the acceptance of the manuscript.

***
Rev_Com_number: RC-2025-03221
New_manu_number: MSB-2025-13511R
Corr_author: Ramirez Flores
Title: ParTIpy: A Scalable Framework for Archetypal Analysis and Pareto Task Inference

The authors addressed the remaining editorial issues.

2nd Apr 2026
Manuscript number: MSB-2025-13511RR
Title: ParTIpy: A Scalable Framework for Archetypal Analysis and Pareto Task Inference

Dear Ricardo,

Thank you again for sending us your revised manuscript. We are now satisfied with the modifications made and I am pleased to inform you that your paper has been accepted for publication.

Your manuscript will be processed for publication by EMBO Press. It will be copy edited and you will receive page proofs prior to publication. Please note that you will be contacted by Springer Nature Author Services to complete licensing and payment information.

You may qualify for financial assistance for your publication charges - either via a Springer Nature fully open access agreement or an EMBO initiative. Check your eligibility: https://link.springer.com/journal/44320/how-to-publish-with-us

Should you be planning a Press Release on your article, please get in contact with embo_production@springernature.com as early as possible in order to coordinate publication and release dates.

If you have any questions, please do not hesitate to contact the Editorial Office. Thank you for your contribution to Molecular Systems Biology.

Kind regards,
Jingyi

Jingyi Hou, PhD
Senior Editor
Molecular Systems Biology

>>> Please note that it is Molecular Systems Biology policy for the transcript of the editorial process (containing referee reports and your response letter) to be published as an online supplement to each paper. If you do NOT want this, you will need to inform the Editorial Office via email immediately. More information is available here: https://link.springer.com/partners/embo-press/editorial-policies#Peer%20review

EMBO Press Author Checklist

Corresponding Author Name: Ricardo Omar Ramirez Flores	
Journal Submitted to: Molecular Systems Biology	
Manuscript Number: MSB-2025-13511-R	

USEFUL LINKS FOR COMPLETING THIS FORM
The EMBO Journal - Author Guidelines
EMBO Reports - Author Guidelines
Molecular Systems Biology - Author Guidelines
EMBO Molecular Medicine - Author Guidelines

Reporting Checklist for Life Science Articles (updated January

This checklist is adapted from Materials Design Analysis Reporting (MDAR) Checklist for Authors. MDAR establishes a minimum set of requirements in transparent reporting in the life sciences (see Statement of Task: 10.31222/osf.io/9sm4x). Please follow the journal's guidelines in preparing your manuscript.

Please note that a copy of this checklist will be published alongside your article.

Abridged guidelines for figures
1. Data
The data shown in figures should satisfy the following conditions:

- the data were obtained and processed according to the field's best practice and are presented to reflect the results of the experiments in an accurate and unbiased manner.
- ideally, figure panels should include only measurements that are directly comparable to each other and obtained with the same assay.
- plots include clearly labeled error bars for independent experiments and sample sizes. Unless justified, error bars should not be shown for technical
- if n<5, the individual data points from each experiment should be plotted. Any statistical test employed should be justified.
- Source Data should be included to report the data underlying figures according to the guidelines set out in the authorship guidelines on Data Presentation.

2. Captions
Each figure caption should contain the following information, for each panel where they are relevant:

- a specification of the experimental system investigated (eg cell line, species name).
- the assay(s) and method(s) used to carry out the reported observations and measurements.
- an explicit mention of the biological and chemical entity(ies) that are being measured.
- an explicit mention of the biological and chemical entity(ies) that are altered/varied/perturbed in a controlled manner.
- the exact sample size (n) for each experimental group/condition, given as a number, not a range;
- a description of the sample collection allowing the reader to understand whether the samples represent technical or biological replicates (including how many animals, litters, cultures, etc.).
- a statement of how many times the experiment shown was independently replicated in the laboratory.
- definitions of statistical methods and measures:

- common tests, such as t-test (please specify whether paired vs. unpaired), simple χ2 tests, Wilcoxon and Mann-Whitney tests, can be unambiguously identified by name only, but more complex techniques should be described in the methods section;

- are tests one-sided or two-sided?
- are there adjustments for multiple comparisons?
- exact statistical test results, e.g., P values = x but not P values < x;
- definition of 'center values' as median or average;
- definition of error bars as s.d. or s.e.m.

Please complete ALL of the questions below.
Select "Not Applicable" only when the requested information is not relevant for your study.

Materials

Newly Created Materials	Information included in the manuscript?	In which section is the information available? (Reagents and Tools Table, Materials and Methods, Figures, Data Availability Section)
New materials and reagents need to be available; do any restrictions apply?	Not Applicable	

Antibodies	Information included in the manuscript?	In which section is the information available? (Reagents and Tools Table, Materials and Methods, Figures, Data Availability Section)
For **antibodies** provide the following information:<br>- Commercial antibodies: RRID (if possible) or supplier name, catalogue number and or/clone number<br>- Non-commercial: RRID or citation	Not Applicable	

DNA and RNA sequences	Information included in the manuscript?	In which section is the information available? (Reagents and Tools Table, Materials and Methods, Figures, Data Availability Section)
**Short novel DNA or RNA including primers, probes**: provide the sequences.	Not Applicable	

Cell materials	Information included in the manuscript?	In which section is the information available? (Reagents and Tools Table, Materials and Methods, Figures, Data Availability Section)
**Cell lines:** Provide species information, strain. Provide accession number in repository **OR** supplier name, catalog number, clone number, and/**OR** RRID.	Not Applicable	
**Primary cultures:** Provide species, strain, sex of origin, genetic modification status.	Not Applicable	
Report if the cell lines were recently **authenticated** (e.g., by STR profiling) and tested for mycoplasma contamination.	Not Applicable	

Experimental animals	Information included in the manuscript?	In which section is the information available? (Reagents and Tools Table, Materials and Methods, Figures, Data Availability Section)
**Laboratory animals or Model organisms:** Provide species, strain, sex, age, genetic modification status. Provide accession number in repository **OR** supplier name, catalog number, clone number, **OR** RRID.	Not Applicable	
**Animal observed in or captured from the field:** Provide species, sex, and age where possible.	Not Applicable	
Please detail **housing and husbandry conditions**.	Not Applicable	

Plants and microbes	Information included in the manuscript?	In which section is the information available? (Reagents and Tools Table, Materials and Methods, Figures, Data Availability Section)
**Plants:** provide species and strain, ecotype and cultivar where relevant, unique accession number if available, and source (including location for collected wild specimens).	Not Applicable	
**Microbes:** provide species and strain, unique accession number if available, and source.	Not Applicable	

Human research participants	Information included in the manuscript?	In which section is the information available? (Reagents and Tools Table, Materials and Methods, Figures, Data Availability Section)
If collected and within the bounds of privacy constraints report on age, sex and gender or ethnicity for all study participants.	Not Applicable	

Core facilities	Information included in the manuscript?	In which section is the information available? (Reagents and Tools Table, Materials and Methods, Figures, Data Availability Section)
If your work benefited from core facilities, was their service mentioned in the acknowledgments section?	Not Applicable	

Design

Study protocol	Information included in the manuscript?	In which section is the information available? (Reagents and Tools Table, Materials and Methods, Figures, Data Availability Section)
If study protocol has been **pre-registered, provide DOI in the manuscript**. For clinical trials, provide the trial registration number **OR** cite DOI.	Not Applicable	
Report the **clinical trial registration number** (at ClinicalTrials.gov or equivalent), where applicable.	Not Applicable	

Laboratory protocol	Information included in the manuscript?	In which section is the information available? (Reagents and Tools Table, Materials and Methods, Figures, Data Availability Section)
Provide DOI OR other citation details if **external detailed step-by-step protocols** are available.	Not Applicable	

Experimental study design and statistics	Information included in the manuscript?	In which section is the information available? (Reagents and Tools Table, Materials and Methods, Figures, Data Availability Section)
Include a statement about **sample size** estimate even if no statistical methods were used.	Not Applicable	
Were any steps taken to minimize the effects of subjective bias when allocating animals/samples to treatment (e.g. **randomization procedure**)? If yes, have they been described?	Not Applicable	
Include a statement about **blinding** even if no blinding was done.	Not Applicable	
Describe **inclusion/exclusion criteria** if samples or animals were excluded from the analysis. Were the criteria pre-established?<br><br>If sample or data points were omitted from analysis, report if this was due to attrition or intentional exclusion and provide justification.	Not Applicable	
For every figure, are **statistical tests** justified as appropriate? Do the data meet the assumptions of the tests (e.g., normal distribution)? Describe any methods used to assess it. Is there an estimate of variation within each group of data? Is the variance similar between the groups that are being statistically compared?	Yes	Figures, Materials and Methods

Sample definition and in-laboratory replication	Information included in the manuscript?	In which section is the information available? (Reagents and Tools Table, Materials and Methods, Figures, Data Availability Section)
In the figure legends: state number of times the experiment was **replicated** in laboratory.	Not Applicable	
In the figure legends: define whether data describe **technical or biological replicates**.	Yes	Figures

Ethics

Ethics	Information included in the manuscript?	In which section is the information available? (Reagents and Tools Table, Materials and Methods, Figures, Data Availability Section)
Studies involving **human participants**: State details of **authority granting ethics approval** (IRB or equivalent committee(s), provide reference number for approval.	Not Applicable	
Studies involving **human participants**: Include a statement confirming that **informed consent** was obtained from all subjects and that the experiments conformed to the principles set out in the WMA Declaration of Helsinki and the Department of Health and Human Services Belmont Report.	Not Applicable	
Studies involving **human participants:** For publication of **patient photos**, include a statement confirming that consent to publish was obtained.	Not Applicable	
Studies involving experimental **animals**: State details of **authority granting ethics approva**l (IRB or equivalent committee(s), provide reference number for approval. Include a statement of compliance with ethical regulations.	Not Applicable	
Studies involving **specimen and field samples:** State if relevant **permits** obtained, provide details of authority approving study; if none were required, explain why.	Not Applicable	

Dual Use Research of Concern (DURC)	Information included in the manuscript?	In which section is the information available? (Reagents and Tools Table, Materials and Methods, Figures, Data Availability Section)
Could your study fall under dual use research restrictions? Please check biosecurity documents and list of **select agents and toxins** (CDC): https://www.selectagents.gov/sat/list.htm	Not Applicable	
If you used a select agent, is the security level of the lab appropriate and reported in the manuscript?	Not Applicable	
If a study is subject to dual use research of concern regulations, is the name of the **authority granting approval and reference number** for the regulatory approval provided in the manuscript?	Not Applicable	

Reporting

The MDAR framework recommends adoption of discipline-specific guidelines, established and endorsed through community initiatives. Journals have their own policy about requiring specific guidelines and recommendations to complement MDAR.

Adherence to community standards	Information included in the manuscript?	In which section is the information available? (Reagents and Tools Table, Materials and Methods, Figures, Data Availability Section)
State if relevant guidelines or checklists (e.g., **ICMJE, MIBBI, ARRIVE, PRISMA**) have been followed or provided.	Not Applicable	
For **tumor marker prognostic studies**, we recommend that you follow the **REMARK** reporting guidelines (see link list at top right). See author guidelines, under 'Reporting Guidelines'. Please confirm you have followed	Not Applicable	
For **phase II and III randomized controlled trials**, please refer to the **CONSORT** flow diagram (see link list at top right) and submit the CONSORT checklist (see link list at top right) with your submission. See author guidelines, under 'Reporting Guidelines'. Please confirm you have submitted this list.	Not Applicable	

Data Availability

Data availability	Information included in the manuscript?	In which section is the information available? (Reagents and Tools Table, Materials and Methods, Figures, Data Availability Section)
Have **primary datasets** been deposited according to the journal's guidelines (see 'Data Deposition' section) and the respective accession numbers provided in the Data Availability Section?	Not Applicable	
Were **human clinical and genomic datasets** deposited in a public access-controlled repository in accordance to ethical obligations to the patients and to the applicable consent agreement?	Not Applicable	
Are **computational models** that are central and integral to a study available without restrictions in a machine-readable form? Were the relevant accession numbers or links provided?	Yes	Material and Methods
If publicly available data were reused, provide the respective **data citations in the reference list**.	Yes	Data Availability Section, Extended View Tables
